# A low-carbon economic dispatch method for regional integrated energy system based on multi-objective chaotic artificial hummingbird algorithm

**DOI:** 10.1038/s41598-024-54733-2

**Published:** 2024-02-19

**Authors:** Jie Cao, Yuanbo Yang, Nan Qu, Yang Xi, Xiaoli Guo, Yunchang Dong

**Affiliations:** 1 School of Computer Science, Northeast Electric Power University, Jilin, 132012 China; 2 Jiangsu Electric Power Co., Ltd. Nanjing Power Supply Company, Nanjing, 210000 China

**Keywords:** Regional integrated energy system, Multi-objective optimization, Low-carbon economic dispatch, Artificial hummingbird algorithm, Computational science, Mathematics and computing, Renewable energy, Energy science and technology, Energy grids and networks

## Abstract

This paper investigates Regional Integrated Energy Systems (RIES), emphasizing the connection of diverse energy supply subsystems to address varied user needs and enhance operational efficiency. A novel low-carbon economic dispatch method, utilizing the multi-objective chaotic artificial hummingbird algorithm, is introduced. The method not only optimizes economic and environmental benefits but also aligns with "carbon peak and carbon neutrality" objectives. The study begins by presenting a comprehensive low-carbon economic dispatch model, followed by the proposal of the multi-objective chaotic artificial hummingbird algorithm, crucial for deriving the Pareto frontier of the low-carbon economic dispatch model. Additionally, we introduce a TOPSIS approach based on combined subjective and objective weights, this approach harnesses the objective data from the Pareto solution set deftly, curbs the subjective biases of dispatchers effectively and facilitates the selection of an optimal system operation plan from the Pareto frontier. Finally, the simulation results highlight the outstanding performance of our method in terms of optimization outcomes, convergence efficiency, and solution diversity. Noteworthy among these results is an 8.8% decrease in system operational economic costs and a 14.2% reduction in carbon emissions.

## Introduction

The evolution of the Energy Internet holds immense promise for catalyzing a radical shift in the energy system. It spearheads the energy revolution and advances the "carbon peak and carbon neutrality" objectives^1^. The regional integrated energy system (RIES) stands as a pivotal embodiment of the Energy Internet. The challenge now is how to tap into its intrinsic economic, low-carbon, flexible, and efficient advantages to address multifaceted operational imperatives, such as elevating the consumption of renewable energy, boosting energy utilization efficiency, and curtailing greenhouse gas emissions^2^.

The pursuit of optimizing Regional Integrated Energy Systems (RIES) to meet diverse operational demands through skillful dispatching techniques is gaining momentum in contemporary research circles^3^. Recent scholarly efforts in RIES optimization and dispatching are increasingly leaning towards multi-objective dispatching, striving to achieve a balance between economic efficiency and low-carbon considerations. Concurrently, heuristic algorithms demonstrate exceptional optimization performance^4,5^, leading to their widespread application in the realm of optimization scheduling for regional integrated energy systems. Abdilahi et al. established an economic dispatch model for integrated energy systems that considers carbon trading costs and applied the Cuckoo Search algorithm for model optimization. The simulation results verified the model's advantages in reducing system carbon emissions^6^. Li et al. proposed an optimization dispatch model for integrated energy systems that considers flexible loads and carbon trading mechanisms. By introducing an emission penalty mechanism, they promoted energy conservation and emission reduction in the system^7^. Wang et al. constructed a ladder-type integrated energy system dispatch model by dividing carbon emission intervals, which took into account both low carbon and economic goals of the system^8^. H et al. transformed a multi-objective optimization problem in a combined cooling, heating, and power system into two single-objective optimization problems, and proposed an improved firefly algorithm to solve the problem, reducing system carbon emissions and economic costs^9^. Zhi et al. established a rural integrated energy system with economic and system stability as optimization goals and used the Simulated Annealing algorithm for optimization, improving system stability ultimately^10^.

While these literary works have shown prowess in transmuting multi-objective optimization challenges into single-objective ones using objective weight coefficients, simplifying model intricacies and refining computational precision, they are not without their pitfalls. The optimization strategies born out of these methods often exhibit a mono-dimensional nature. Precursors like weight coefficients for various objectives remain elusive, resulting in the final optimization outcomes being overly reliant on dispatchers' subjective expertise. This hampers the system's agility to adapt to diverse objectives, such as operational economy and low carbon, under varying scenarios.

Currently, multi-objective optimization algorithms are gaining traction in RIES dispatch problems due to their ability to optimize multiple objectives concurrently. By doing so, they yield the Pareto optimal frontier, offering dispatchers a diverse range of choices that cater to various goals. Abuelrub et al. combined the development capability of biogeography-based optimization (BBO) and the exploration ability of particle swarm optimization (PSO) to propose a Greedy Particle Swarm and biogeography-based optimization algorithm (GPSBBO), which they applied to solve the multi-objective optimization design of integrated energy systems with energy storage^11^. Wu et al. proposed the multi-objective non-dominated sorting genetic algorithm (MO-NSGA-II) algorithm and used it to optimize the multi-objective optimization model of the constructed electricity-heat regional integrated energy system, improving the exergic efficiency and reducing pollution emissions of system operation^12^. Nazari et al. introduced a novel multi-objective optimization algorithm termed as the multi-objective multi-verse optimizer (MOMVO). This algorithm aims to enhance exergy efficiency while concurrently minimizing the system's product cost rate. Comprehensive evaluations of the system were conducted through energy, exergy, and exergo-economic perspectives^13^. Wang et al. embedded the Tabu search algorithm (TSA) into the multi-objective genetic algorithm, proposing a multi-objective hybrid optimization (MOHO) algorithm, and used this method to solve the dual-layer optimization model of the regional integrated energy system, effectively reducing the system's economic cost while also improving the system's efficiency^14^. Patwal et al. combined a pumped-storage hydrothermal system with wind energy, solar energy, and battery units, using economic cost and environmental cost as optimization objectives, and applied an improved crossover particle swarm optimization (ICPSO) for optimization. The Pareto optimal solutions have proven the energy-saving and emission reduction benefits of the suggested model^15^. Wang et al. devised an integrated energy system optimization model, rich in renewable energy, and introduced a scenario dominance-based multi-objective evolutionary algorithm (MOEA) to bolster both the system's economics and stability^16^. Liu et al. advanced a multi-objective gravitational search optimization (MOGSO) algorithm, leading to diminished energy consumption in the assessed system^17^. Li et al. integrated a multi-objective whale optimization (MOWO) algorithm to solve their dual-layer robust game model for the regional energy system, witnessing improvements in the system's economic performance and dispatching adaptability^18^.

In summary, the utilization of multi-objective optimization algorithms presents solutions to the intricate scheduling challenges faced by regional integrated energy systems. The resulting Pareto frontier furnishes dispatchers with a spectrum of options for the ultimate scheduling blueprint. However, challenges endure. Some algorithms are susceptible to getting trapped in local optima, especially when dealing with complex, constraint-laden problems^19^. Additionally, there may be a shortfall in global search capabilities, warranting improvements in their overall performance^20^. These limitations lead to extended problem-solving durations and less-than-ideal optimization outcomes.

In essence, multi-objective optimization algorithms present viable solutions to the scheduling challenges of regional integrated energy systems. The derived Pareto frontier offers dispatchers an array of choices for finalizing the scheduling strategy. Nevertheless, a subset of these algorithms is susceptible to entrapment in local optima, especially when faced with intricate constraints. Others lack robust global search capabilities. The efficacy of such algorithms requires enhancement, as their current limitations result in protracted problem-solving durations and less-than-optimal optimization outcomes. To advance the "dual carbon" objectives and address existing challenges in achieving low-carbon economic operation within regional integrated energy systems, this paper builds upon existing research. It presents a low-carbon economic dispatch model for these systems. Moreover, an optimization scheduling technique rooted in the multi-objective algorithm is introduced for model resolution. The key contributions of our work include.

1. A holistic optimization model for the electricity-heat-gas subsystems within the regional integrated energy system is established, accommodating both its economic and environmental dimensions. This model accounts for the system's topology and inherent constraints.
2. A novel multi-objective chaotic artificial hummingbird strategy is introduced, integrating chaotic mapping and dynamic adjustments. Utilizing non-dominance and congestion distance metrics, this strategy refines the initial model. It pinpoints the optimal Pareto boundary for the RIES low-carbon economic dispatch challenge.
3. We introduce a TOPSIS approach based on subjective and objective combined weights. From the obtained Pareto frontier, the system's final dispatch plan that meets the system's operating requirements is selected. This is done to achieve low-carbon economic dispatching of the system.
4. Through rigorous simulation, the efficacy of our proposed methodology is validated. The results underline MOCAHA's superior capacity in addressing the RIES low-carbon economic conundrum across optimization outcomes, convergence efficiency, and diverse solution sets. This approach amplifies both the economic and environmental dividends of RIES. Concurrently, the proposed TOPSIS based on subjective and objective combined weights minimizes potential errors from dispatcher experiences, delivering a precise and holistic system dispatching plan.

The subsequent sections of this paper are organized as follows: In “Introduction”, we delve into the foundational architecture of the regional integrated energy system, outline the low-carbon economic dispatch model, and lay out the defined objective functions and constraints pivotal to system optimization. “Low-carbon economic dispatch model for RIES” offers an in-depth elucidation of the introduced multi-objective optimization scheduling methodology. “Low-carbon economic dispatch method for RIES based on MOCAHA” showcases a simulation experiment performed on a test node and provides a thorough dissection and interpretation of the yielded results. Finally, “Case study” wraps up the primary findings of this paper and delineates potential avenues for forthcoming research.

## Low-carbon economic dispatch model for RIES

**Figure 1:**
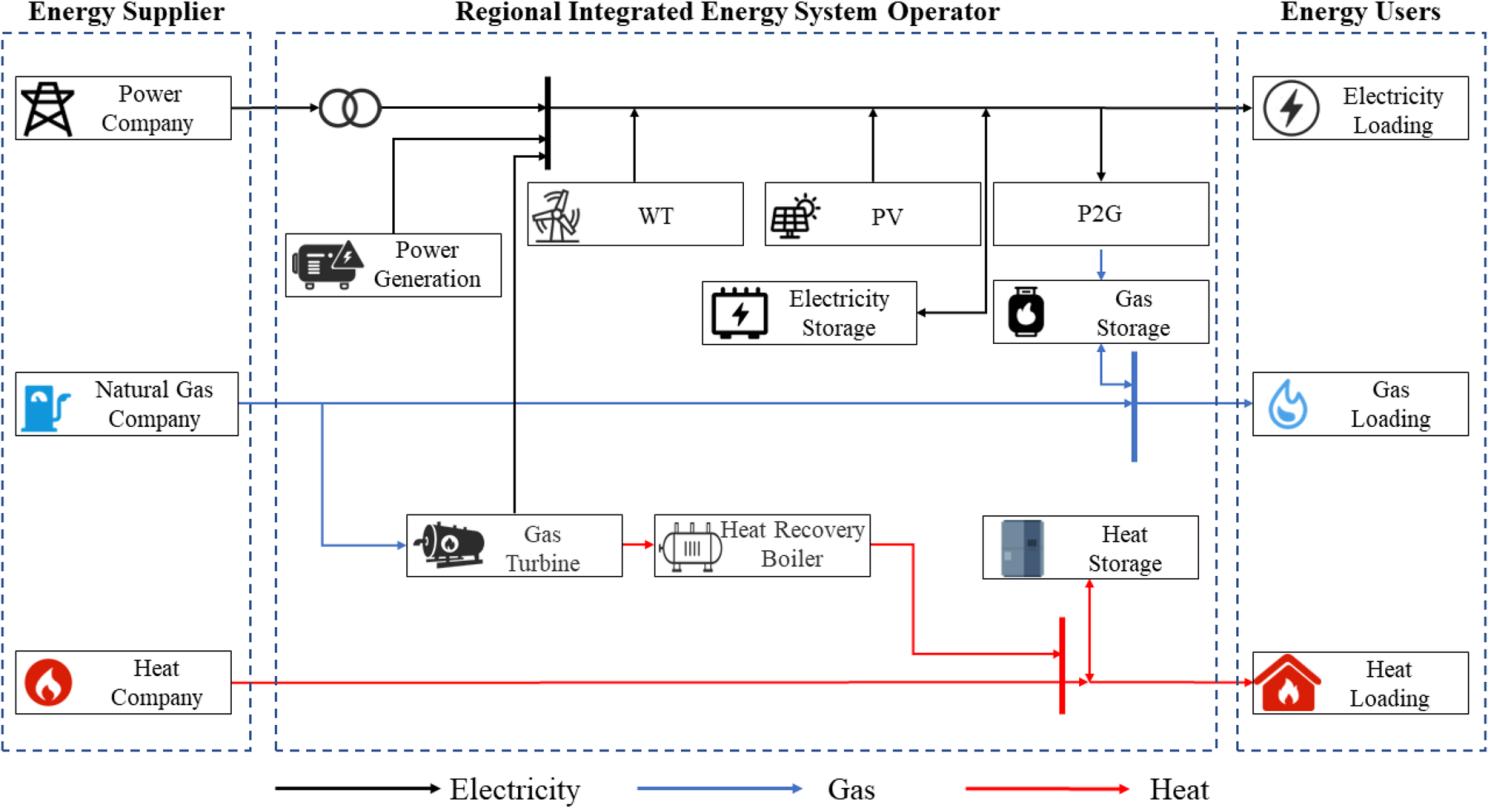
Overall framework of the regional integrated energy system.

The regional integrated energy system (RIES) stands as a sophisticated energy coupling mechanism, bridging the gap between user levels and expansive cross-regional integrated energy systems. This system epitomizes the trajectory of future energy internet advancements^21^. Comprising three primary entities, energy suppliers, RIES operators, and integrated energy users, the ecosystem of RIES is robust. Energy suppliers encompass entities like power companies, natural gas corporations, and heating agencies. RIES operators work diligently to satiate the diverse energy needs of integrated users, such as electricity, heat, and gas. They do this by orchestrating energy output from the internal subsystems and sourcing energy from the aforementioned suppliers. A visual representation of the RIES framework can be gleaned from Fig. 1.

Within the RIES construct, myriad energy supply subsystems intertwine, facilitated by energy transformation devices like gas turbines, combined heat and power (CHP) units, and power-to-gas (P2G) devices. In RIES, user electricity loads are met by equipment such as coal-fired generators, gas turbines, wind turbine generators, and photovoltaic cells, with energy transactions occurring between the system and the external power grid. The heat produced by gas-fired boilers is used to meet user heat load requirements. CHP units serve as coupling devices between the power supply subsystem and the heat supply subsystem, providing both electricity and heat to users within the system, achieving the coupling of the power supply and heat supply subsystems. Meanwhile, P2G equipment captures CO_2_ from the flue gas of fossil fuel combustion and synthesizes methane for gas units, realizing the coupling of the power supply and gas supply subsystems.

By orchestrating the harmonized operation of RIES, we can enhance energy utilization efficiency, curtail energy consumption, and mitigate environmental impacts. Such coordination culminates in notable economic and environmental dividends.

### Equipment operating characteristics

Optimizing the regional integrated energy system necessitates a thorough understanding of each equipment unit's operational characteristics. The aim is to ensure that every energy-producing unit's output aligns with the system's load demand. Consequently, to maximize the energy efficiency of the RIES, we've formulated mathematical models for key energy-producing units, including coal-fired generators, combined heat and power units, wind turbines, and other representative equipment.

1. Coal-fired Power Generation Unit.
  The coal consumption characteristics of coal-fired power generation units are inherently nonlinear. In practical applications, a second-order approximation model is usually used. The coal consumption of coal-fired power generation units is as shown in Eq. (1).
  1 $$F_{{{\text{G,}}t}} = \sum\limits_{i \in \Omega } {\alpha_{i} \left( {P_{i,t} } \right)^{2} + \beta_{i} P_{i,t} + \gamma_{i} }$$
  where *F*_*G,t*_ is the coal consumption of coal-fired power generation unit *i* at time period *t*, *P*_*i,t*_ is the actual active power output of coal-fired power generation unit *i* at time period *t*, and *α*_*i*_, *β*_*i*_, and *γ*_*i*_ are the coal consumption coefficients of coal-fired power generation unit *i*, Ω is the set of coal-fired power generation unit.
2. Combined Heat and Power Unit.
  The fuel consumption of a combined heat and power (CHP) unit, which can use coal, natural gas, or other fuels to provide both electricity and heat to the system's users, is related to its output of electrical power and thermal power. The fuel consumption characteristics are shown in Eq. (2).
  2 $$F_{CHP,t} = \sum\limits_{{i \in \Omega_{CHP} }} {a_{CHP,i} \left( {P_{i,t}^{CHP} } \right)^{2} + b_{CHP,i} P_{i,t}^{CHP} + c_{CHP,i} \left( {H_{i,t}^{CHP} } \right)^{2} + d_{CHP,i} H_{i,t}^{CHP} + e_{CHP,i} P_{i,t}^{CHP} H_{i,t}^{CHP} + f_{CHP,i} }$$
  where *F*_*CHP,t*_ is the fuel consumption of the CHP unit *i* at time period *t*, *P*_*i,t*_^*CHP*^ is the active power output of the CHP unit *i* at time period *t*; *H* is the thermal output of the CHP unit *i* at time period *t*; *a*_*CHP,i*_, *b*_*CHP,i*_, *c*_*CHP,i*_, *d*_*CHP,i*_, *e*_*CHP,i*_, and *f*_*CHP,i*_ are the fuel consumption coefficients of the CHP unit *i*; Ω is the set of CHP units.
3. Wind Power Generation Unit.
  The active power of wind power generators is influenced by factors such as the availability of wind energy, wind speed, wind turbine power curves, wind turbine shape, and turbine size. We use a simplified mathematical model for wind power generators, representing the active power of wind power generators as a piecewise function related to the wind speed of the wind farm at that time period. The forecasted power output of the wind power generator is shown in Eq. (3).
  3 $$P_{w,t}^{forecasted } = \left\{ {\begin{array}{*{20}l} 0 \hfill & {,V_{t} > V^{CO} ,V_{t} < V^{CI} } \hfill \\ {P^{\max } \times \left( {\frac{{V_{t} - V^{CI} }}{{V^{R} - V^{CI} }}} \right)} \hfill & {,V^{CI} \le V_{t} < V^{R} } \hfill \\ {P^{\max } } \hfill & {,V^{R} \le V_{t} \le V^{CO} } \hfill \\ \end{array} } \right.$$
  where *P*_*w,t*_ is the forecasted active power output of the wind power generator at time period *t*; *P*_*max*_ is the maximum power output of the wind power generator; *V*^*CO*^, *V*^*CI*^, and *V*^*R*^ represent the cut-out wind speed, cut-in wind speed, and rated wind speed of the wind power generator, respectively; *V*_*t*_ is the forecasted wind speed of the wind farm at time period *t*.

### Objective functions

Based on the equipment operating features outlined in “Equipment operating characteristics”, we've developed a low-carbon economic dispatch model for RIES. The model encompasses two primary objectives: minimizing system operational costs and carbon emissions. The goal is to optimize both the system's total operational expenses and its carbon footprint harmoniously.

1. Economic Cost.
  The economic cost of RIES operation mainly considers factors such as the energy consumption cost of energy conversion equipment, the cost of purchasing energy from external energy networks, and the penalty for abandoning renewable energy. The details are given in Eq. (4).
  4 $$\left\{ \begin{gathered} \min Cost_{all} = Cost_{units} + Cost_{buy} + Cost_{PEN} \hfill \\ Cost_{units} = \sum {F_{unit} \times Price_{f} + P_{unit} \times \mu_{unit} } \hfill \\ Cost_{buy} = Cost_{buy,P} + Cost_{buy,H} + Cost_{{buy,{\text{G}}}} \hfill \\ Cost_{PEN} = Cost_{PEN,imb} + Cost_{PEN,waste} \hfill \\ \end{gathered} \right.$$
  where *Cost*_*all*_, *Cost*_*units*_, *Cost*_*buy*_, and *Cost*_*PEN*_ represent the total economic cost of system operation, the total cost of operating and maintaining units in the system, the total amount of energy purchase transactions of the system, and the operation penalty, respectively.
  *F*_*unit*_, *Price*_*f*_, *P*_*unit*_, *μ*_*unit*_ refer to the system's fuel consumption, unit price of fuel, power output of each unit, and unit operation and maintenance cost, respectively. *Cost*_*buy,P*_, *Cost*_*buy,H*_, *Cost*_*buy,G*_ refer to the system's cost of purchasing electricity, heat, and gas, respectively. *Cost*_*PEN,imb*_ and *Cost*_*PEN,waste*_ represent the penalty for power imbalance and energy waste, respectively.
2. Carbon Emissions.
  The carbon emissions of RIES mainly consider the carbon emissions generated by energy conversion equipment and the equivalent carbon emissions of the energy purchased from external energy networks, as shown in Eq. (5).
  5 $$\left\{ \begin{gathered} \min E_{all} = E_{units} + E_{buy} \hfill \\ E_{units} = F_{units} \times E_{f} \hfill \\ E_{buy} = P_{buy} \times E_{buy,P} + H_{buy} \times E_{buy,H} + G_{buy} \times E_{buy,G} \hfill \\ \end{gathered} \right.$$
  where *E*_*all*_, *E*_*units*_, *E*_*buy*_, *E*_*f*_, *E*_*buy,P*_, *E*_*buy,H*_, and *E*_*buy,G*_ represent the total carbon emissions of the regional integrated energy system, carbon emissions from unit operation, equivalent carbon emissions from energy purchases, carbon emissions per unit of fuel, equivalent carbon emissions from electricity purchases, equivalent carbon emissions from heat purchases, and equivalent carbon emissions from gas purchases, respectively. *F*_*units*_ represent the fuel consumption of the system's units. *P*_*buy*_, *H*_*buy*_, and *G*_*buy*_ represent the system's electricity, heat, and gas purchases, respectively.

### Constraints

To maintain the safe and reliable operation of the regional integrated energy system, each unit's functioning is governed by certain constraints. These include the balance between supply and demand, equipment operations, and system power flow. Leveraging the operational characteristics described in “Equipment operating characteristics”, we classify system constraints according to their respective subsystems. Specifically, these constraints fall into three categories: those of the electricity subsystem, the natural gas subsystem, and the thermal subsystem. The following section elaborates on the constraints specific to each subsystem.

1. Electricity Subsystem.
  The constraints on the operation of the electrical grid in a regional integrated energy system primarily include power balance constraints, unit output constraints, unit ramp-up constraints, and system power flow constraints, as shown in Eq. (6).
  6 $$\left\{ \begin{gathered} P_{units,t} + P_{buy,t} = P_{load,t} \hfill \\ P_{\min ,i,t} \le P_{i,t} \le P_{\max ,i,t} \hfill \\ 0 \le P_{W,t} \le P_{f,W,t} \hfill \\ P_{i,t} - P_{i,t - 1} \le R_{i,U} \hfill \\ P_{i,t - 1} - P_{i,t} \le R_{i,D} \hfill \\ \underline{P}_{ij} \le P_{ij,t} \le \overline{P}_{ij} \hfill \\ \end{gathered} \right.$$
  where *P*_*units,t*_ is the output power of coal-fired power units, CHP units, wind power units, and other units at time *t*; *P*_*load,t*_ is the system's electricity demand at time t; *P*_*max,i,t*_ and *P*_*min,i,t*_ are the upper and lower output limits of unit *i*, respectively; *R*_*i,U*_ and *R*_*i,D*_ are the ramp-up and ramp-down rates of unit *i,* respectively; *P*_*ij,t*_ is the power flow value of branch *ij* at time *t*; $\underline{P}_{ij}$, $\overline{P}_{ij}$ are the lower and upper power flow limits of branch *ij*, respectively.
2. Gas Subsystem.
  The operating constraints of the gas network in the RIES mainly include gas flow balance constraints, gas flow constraints, etc., as shown in Eq. (7).
  7 $$\left\{ \begin{gathered} w_{k}^{\min } \le w_{k,t} \le w_{k}^{\max } \hfill \\ f_{mn}^{\min } \le f_{mn,t} \le f_{mn}^{\max } \hfill \\ \end{gathered} \right.$$
  where *w*_*k*_^*min*^ and *w*_*k*_^*max*^ represent the upper and lower limits of gas supply from the gas well, respectively. *w*_*k*,t_ denotes the gas supply from gas well *k* in period *t*. *f*_*mn*_^*min*^, *f*_*mn*_^*max*^ represent the upper and lower limits of pipeline gas flow, respectively. *f*_*mn,t*_ indicates the gas flow in pipeline *mn* in period *t*.
3. Thermal Subsystem.
  The operational constraints of the RIES's heat network mainly include heat network power balance constraints and unit output constraints, as shown in Eq. (8).
  8 $$\left\{ \begin{gathered} H_{{{\text{buy}},t}} + H_{CHP,t} = H_{load,t} \hfill \\ H_{min,CHP}^{{}} \le H_{CHP,t} \le H_{max,CHP} \hfill \\ H_{i,t}^{ \, } - H_{i,t - 1}^{ \, } \le R_{i,U} \hfill \\ H_{i,t - 1}^{ \, } - H_{i,t}^{ \, } \le R_{i,D} \hfill \\ \end{gathered} \right.$$
  where *H*_*buy,t*_ is the purchased heating power at time *t*; *H*_*CHP,t*_ is the heat output power of the CHP unit at time; *H*_*load,t*_ is the heat load demand of the system at time *t*; *H*_*max,CHP*_ and H_min,CHP_ are the upper and lower limits of the heat output power of the CHP unit, respectively; *R*_*i,U*_ and *R*_*i,D*_ are the ramp-up and ramp-down rates of unit *i*, respectively.

To summarize, in our pursuit of low-carbon and economically efficient dispatch within RIES, we've delineated a topology for electricity-heat-gas systems. Decision variables are represented by the power outputs from equipment like coal-fired power units, wind power units, and CHP units over varying time intervals. We 've designated Eq. (4) for economic objective and Eq. (5) for environmental objective. Furthermore, Eqs. (6)–(8) serve as the system's operational constraints. Through these measures, we've constructed an RIES dispatch model that harmoniously integrates both economic and environmental considerations in its operation.

## Low-carbon economic dispatch method for RIES based on MOCAHA

The RIES low-carbon economic dispatch challenge is inherently intricate and non-convex. Its solution domain is typically nonlinear, discrete, and replete with multiple local optima. Confronted with high-constraint RIES scheduling, some optimization algorithms are susceptible to entrapment within these local optima, hindering the global optimal solution's identification. Moreover, various optimization strategies exhibit diminished global search capabilities, resulting in an incomplete traversal of the solution landscape and potentially overlooking the true optimum. Such shortcomings can culminate in protracted problem-solving durations, inhibiting timely solution attainment. Even when solutions are found, they might stray from the optimal considerably. Intrinsically, the RIES low-carbon economic dispatch is a multi-objective conundrum. Contemporary dispatching approaches, in tackling multi-objective dilemmas, often resort to subjective weighting mechanisms. Such methods entail schedulers assigning weights to distinct objectives grounded in their expertise and discretion. Nonetheless, this methodology is not without pitfalls. It is inherently reliant on the dispatcher's subjective experience, potentially yielding inconsistent outcomes. Furthermore, it may fall short in encapsulating the intricate interrelations and dependencies among objectives, rendering the pursuit of an authentically optimal outcome arduous.

**Figure 2:**
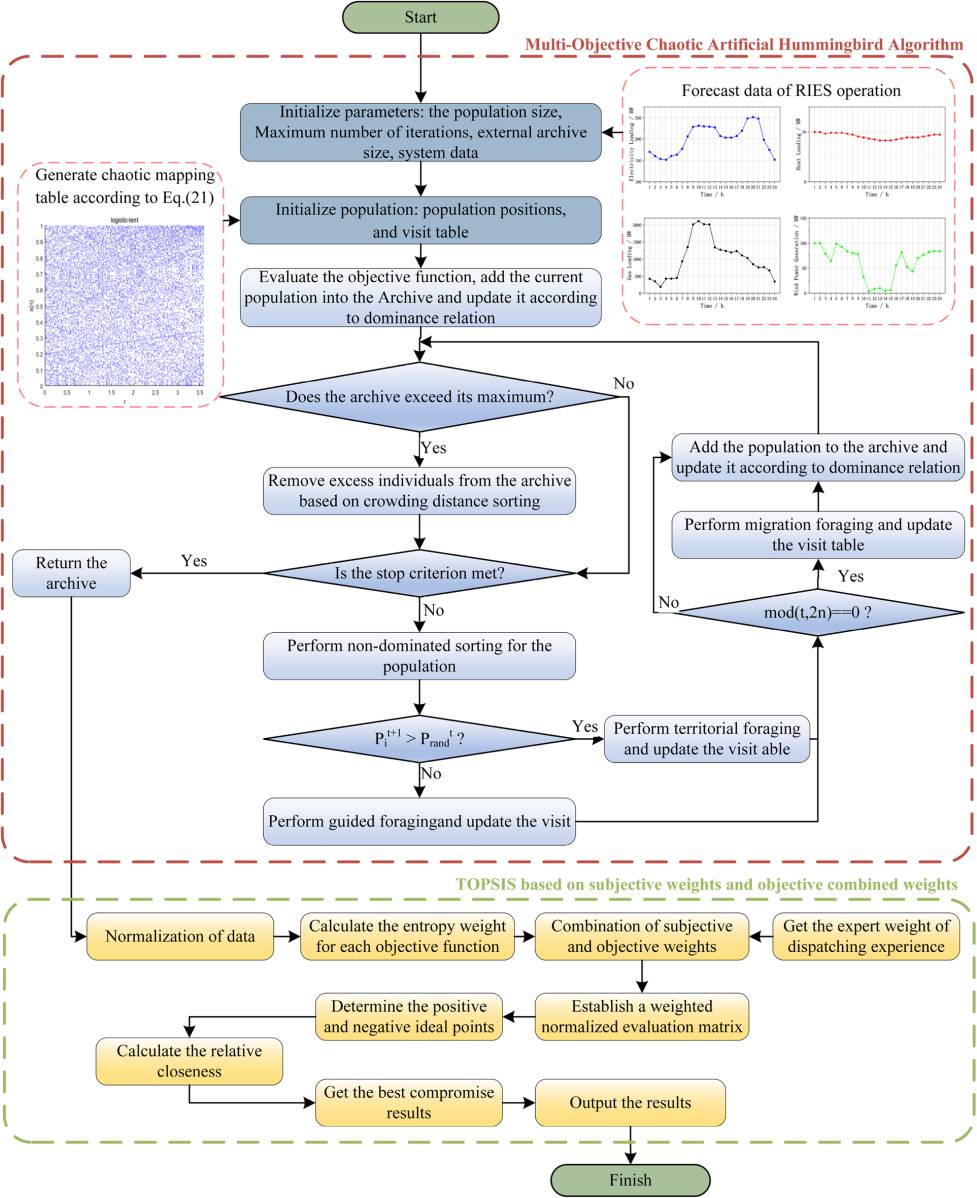
Flowchart of the RIES Low-carbon economic dispatch method.

The performance and outcome of a scheduling plan can be severely impacted by the issues previously discussed, which subsequently influence the overall operational efficiency of the energy system. Addressing these challenges, we introduce a novel RIES low-carbon economic scheduling approach designed to enhance the coordinated optimization of the RIES system. This aims to reduce both operational costs and carbon emissions. The complete procedure of this proposed method is illustrated in Fig. 2.

Our method incorporates two main components tailored to solve the RIES low-carbon economic scheduling conundrum. Firstly, we focus on the application and refinement of AHA, resulting in the development of the multi-objective chaotic artificial hummingbird algorithm. Once the RIES operational data is fed into this algorithm, it conducts a thorough global search in the optimization space. This exhaustive search considers the reduction of economic and carbon emissions simultaneously. The outcome of this process is a suite of Pareto optimal solutions, constituting the Pareto frontier for the RIES economic dispatch issue.

The second component revolves around the application and enhancement of the Technique for Order Preference by Similarity to an Ideal Solution (TOPSIS). Specifically, we have incorporated the TOPSIS approach that merges both subjective and objective criteria (TOPSIS-SOCW). This strategy evaluates the objective attributes of the Pareto frontier using the entropy weight method and simultaneously integrates the insights and judgment of system scheduling experts. As a result, TOPSIS-SOCW offers a holistic ranking of all potential solutions within the Pareto frontier. This culminates in the identification of an optimal solution, balancing economic and low-carbon priorities, forming the finalized operational plan for RIES. In essence, this method combined two complementary algorithmic steps addresses the RIES economic dispatch challenge adeptly, ensuring the system's optimal performance.

### Traditional artificial hummingbird algorithm

Optimization algorithms play a pivotal role in addressing the RIES scheduling challenge. They assist decision-makers in identifying operation plans that adhere to system operational constraints and optimize the system's objectives. Among these algorithms, AHA stands out due to its inherent flexibility in parameter settings. Notably, it demands fewer adjustable parameters compared to many of its counterparts. This streamlined configuration not only facilitates the ease of implementation and debugging but also mitigates the potential for performance drop-offs stemming from unsuitable parameter choices. Additionally, AHA is designed with effective exploration and exploitation techniques, enabling it to rapidly pinpoint promising solutions within an expansive search domain and delve into these solutions for refined results deeply. Crucially, AHA possesses the capability to navigate complex, non-convex terrains without frequently succumbing to local optima traps. Collectively, these attributes render AHA especially apt for tackling RIES optimization scheduling issues.

The Artificial Hummingbird Algorithm (AHA) is a novel meta-heuristic optimization algorithm proposed by Zhao et al., inspired by the nectar-foraging behavior of hummingbirds^22,23^. What sets hummingbirds apart is their astonishing foraging memory. Hummingbirds have a hippocampus in their brains that is much larger than that of any other bird, playing a critical role in learning and memory. AHA simulates three specific flight skills of hummingbirds in nature, namely axial flight, diagonal flight, and omnidirectional flight, as well as three intelligent foraging strategies: guided foraging, regional foraging, and migratory foraging. By introducing a visit table, AHA realizes the memory function of hummingbirds in finding and selecting food sources, ultimately achieving the goal of solving optimization problems.

In AHA, *L* hummingbirds move in a *D*-dimensional search space to find the optimal solution for the problem to be optimized. The position of each hummingbird individual is the food source it visits, representing a feasible solution to the problem being optimized, denoted as $X_{i} = (x_{i,1} ,x_{i,2} ,...,x_{i,D} )$. The nectar replenishment rate of the food source represents the fitness value corresponding to the feasible solution. The AHA algorithm steps are as follows:

***Step 1:*** Initialization.

AHA places *n* hummingbirds on *n* food sources. The positions of the food sources are randomly initialized according to Eq. (9):

9 $$x_{i} = S_{{\text{L}}} + r \times \left( {S_{{\text{u}}} - S_{{\text{L}}} } \right),i = 1,2,...,n$$

*x*_*i*_ represents the position of the *i*th food source;* n* represents the population size; *S*_*u*_ and *S*_*L*_ represent the upper and lower limits of the search space, respectively; *r* represents a random number uniformly distributed between [0, 1].

The food source visitation table is initialized according to Eq. (10):

10 $$V_{i,j} = \left\{ {\begin{array}{*{20}c} {0,} & {{\text{if}}} \\ {{\text{null}},} & {{\text{if}}} \\ \end{array} } \right.\begin{array}{*{20}c} {i \ne j} \\ {i = j} \\ \end{array} ,i = 1,2,...,n;j = 1,2,...,n$$

where *V*_*i,j*_ is the numerical value of row *i* and column *j* of visit table V; *i* = *j* means the hummingbird is feeding at a specific food source; *i* ≠ *j* indicates that the *j*th food source has been visited by the *i*th hummingbird in the current iteration.

***Step 2:*** Guided foraging.

In order to obtain more nectar, hummingbirds will visit the food source with the highest nectar replenishment rate among food sources at the same visit level. During the foraging process, a direction switching vector is introduced to describe three skills: axial flight, diagonal flight, and omnidirectional flight.

These flight patterns can be extended to a d-D space. The axial flight is defined as shown in Eq. (11):

11 $$D^{(i)} = \left\{ {\begin{array}{*{20}c} {1,} & {{\text{if }}i = {\text{rand}}(\left[ {1,d} \right])} \\ {0,} & {{\text{else}}} \\ \end{array} } \right.$$

The diagonal flight is defined as shown in Eq. (12):

12 $$D^{(i)} = \left\{ {\begin{array}{*{20}c} {1,} \\ {0,} \\ \end{array} \, \begin{array}{*{20}c} {{\text{if }}i = P(j),j \in [1,k],P = randperm(k),k \in \{ 2,[r_{1} \times (d - 2) + 1]\} } \\ {else} \\ \end{array} } \right.$$

The omnidirectional flight is defined as shown in Eq. (13):

13 $$D^{(i)} { = 1}$$

where *D*^*(i)*^ represents the flying skill; *rand*([1, *d*]) indicates generating a random integer from 1 to *d*; *randperm*(*k*) denotes creating a random permutation of integers from 1 to *k*; *r*_*1*_ represents a random number uniformly distributed between (0, 1]; *d* represents the dimension of the problem. Where *i* = 1, 2, …, *d*.

The mathematical descriptions of these flying skills allow hummingbirds to visit target food sources and obtain candidate food sources. The update of the position of the candidate food source is mathematically described as follows in Eq. (14):

14 $$v_{i} (t + 1) = x_{i,tar} (t) + a \times D \times \left[ {x_{i} (t) - x_{i,tar} (t)} \right]$$

where *v*_*i*_(*t* + *1*) represents the position of the *i*th candidate food source at iteration *t* + *1*; *x*_*i*_(*t*) represents the position of the *i*th food source at iteration *t*; *x*_*i,tar*_(*t*) represents the position of the target food source that the *i*th hummingbird will visit; and *a* is a guided factor, which is subject to the normal distribution with mean = 0 and standard deviation = 1.

The position update of the *i*th food source during guidance foraging is shown in Eq. (15):

15 $$x_{i} (t + 1) = \left\{ {\begin{array}{*{20}c} {x_{i} (t),} & {f[x_{i} (t)] \le f[v_{i} (t + 1)]} \\ {v_{i} (t + 1),} & {f[x_{i} (t)] > f[v_{i} (t + 1)]} \\ \end{array} } \right.$$

where *x*_*i*_(*t* + *1*) represents the position of the *i*th food source at iteration (*t* + 1); *f(x)* represents the fitness value of the function; other parameters have the same meanings as above.

***Step 3:*** Territorial foraging.

After visiting the target food source, hummingbirds are likely to move to nearby areas outside their territory to search for new food sources rather than visit other existing food sources. The mathematical description of the position update of the candidate food source in the nearby area is shown in Eq. (16):

16 $$v_{i} (t + 1) = x_{i} (t) + b \times D \times x_{i} (t)$$

where *b* is the territorial factor, which is subject to a normal distribution with mean equals 0 and standard deviation equals 1; other parameters have the same meanings as above.

***Step 4:*** Migration foraging.

When the area frequently visited by hummingbirds is lacking in food, hummingbirds usually migrate to more distant food source areas to forage. The mathematical description of the position update of the food source with the worst nectar replenishment rate is shown in Eq. (17):

17 $$x_{{{\text{wor}}}} (t + 1) = S_{{\text{L}}} + r \times (S_{u} - S_{{\text{L}}} )$$

where *x*_*wor*_*(t* + *1)* is the position of the food source with the worst nectar replenishment rate in the population at iteration *(t* + *1)*; other parameters have the same meanings as above.

### Optimization method of low-carbon economy based on multi-objective chaotic artificial hummingbird algorithm

The traditional artificial hummingbird algorithm targets single-objective optimization challenges predominantly. When confronted with multi-objective optimization dilemmas, it struggles to harmonize conflicting optimization goals^22^. Addressing this shortcoming, we've instilled a multi-objective optimization framework that leans on non-dominated sorting and crowding distance sorting. By leveraging chaotic mapping, we generate the initial population, and a dynamic adjustment approach is integrated to bolster the algorithm's optimization prowess. Building on these enhancements, we advocate for the multi-objective chaotic artificial hummingbird algorithm (MOCAHA) as an apt solution for the low-carbon economic operational quandary present in regional integrated energy systems. The specific steps of MOCAHA are as follows:

***Step 1***: Input the forecasted load and equipment parameters for the RIES system and initialize the relevant parameters.

***Step 2***: Initialize the hummingbird population using the Logistic-Tent chaotic mapping method.

***Step 3***: Evaluate the objective functions for all food sources, followed by non-dominated sorting, where each food source corresponds to a system operation scheme.

***Step 4***: Incorporate the sorted operation schemes into the external archive and utilize the crowding distance sorting algorithm to maintain the Archive.

***Step 5***: Perform operations such as guided foraging, regional foraging, and migration foraging for all hummingbird individuals based on their dominance rank and the number of iterations. Update the visitation table accordingly.

***Step 6***: Iterate through ***Step 3*** to ***Step 5*** until the termination conditions are met and subsequently output the final Archive. The external archive comprises a series of economical and low-carbon operation schemes.

#### Construction of multi-objective artificial hummingbird algorithm

The challenge of optimizing low-carbon economic operations within RIES fundamentally hinges on multi-objective optimization. Contrastingly, the traditional AHA primarily functions as a single-objective optimization algorithm. To adapt AHA for this multifaceted issue, we've integrated a non-dominated sorting algorithm^24^. Alongside this, we've incorporated an external archive, rooted in the crowding distance sorting method, culminating in the development of a multi-objective artificial hummingbird algorithm.

Firstly, the non-dominated sorting algorithm was integrated to ascertain the dominance level of each RIES optimization scheduling solution. Subsequently, an external archive grounded in the crowding distance sorting approach was incorporated. Throughout the algorithm's iterative phase, optimal non-dominated solutions are consistently retained within these external archives. To effectively manage the archive size during the optimization progression, a crowding distance-based sorting methodology is utilized, which serves to enhance both solution diversity and the convergence speed of the population.

1. Non-dominated Sorting.
  The core idea of the non-dominated sorting is to rank all solutions based on their dominance relationships in the objective space. Dominance relation means that one solution is better than another solution in all objectives, or at least better in one objective.
  For the multi-objective optimization problem of RIES, for an optimization problem with *n* objective functions (where *n* is a positive integer), there is a equation as follows:
  18 $$\left\{ {\begin{array}{*{20}c} {f_{i} (X_{a} ) \ge f_{i} (X_{b} ),\forall i = 1,2,...,n} \\ {f_{i} (X_{a} ) > f_{i} (X_{b} ),\exists i = 1,2,...,n} \\ \end{array} } \right.$$
  where *X*_*a*_ and *X*_*b*_ are any two given scheduling solutions, and *f*_*i*_*(x)* represents the *i*th objective function fitness.
  If Eq. (18) holds, then *X*_*b*_ is said to dominate *X*_*a*_. By comparing the dominance relationships of all solutions, they can be divided into different dominance levels F(F = 1, 2, …, n). Figure 3 shows a schematic diagram of non-dominated sorting with two objectives. If a decision variable is not dominated by any other decision variable, it is called a non-dominated solution. All non-dominated solutions in the solution set form the Pareto solution set.

**Figure 3:**
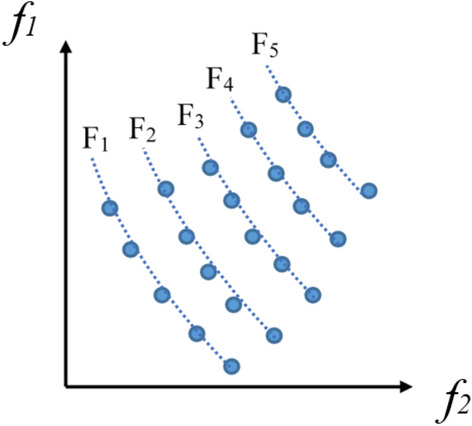
Schematic diagram of non-dominated sorting under two objectives.
  The non-dominated sorting can achieve a reasonable balance between multiple conflicting optimization objectives. At the same time, the dominance level of each operation plan obtained can show the importance of each scheduling plan in the current population, providing an important basis for the dynamic adjustment of algorithm factors in “Dynamic adjustment factor”.
2. The External Archive Based on Crowding Distance Sorting.
  We introduced an external archive based on crowding distance sorting, and after each iteration, we stored the operation plans in the Pareto solution set obtained through non-dominated sorting into the external archive^25^. At the same time, as the scheduling plans in the external archive will change continuously with the progress of iteration, we use the crowding distance sorting algorithm to maintain the size of the external archive, to enhance the diversity of the population within the external archive, avoid the optimization process falling into local optimal solutions prematurely, and provide more choices and stronger decision-making basis for subsequently selecting the optimal compromise scheduling plan from the Pareto front.
  The crowding distance sorting is a non-parametric method that can enhance the diversity of the Pareto solution set. It maintains a fixed-size external archive by removing overly redundant solutions with smaller crowding distances. The specific steps are as follows:
  ***Step1***: For each individual in the Pareto front, initialize their crowding distance to 0.
  ***Step2***: For each objective function, sort the individuals according to that objective function. Set the crowding distances of the first and last individuals in the sorted list to infinity, indicating their boundary positions.
  ***Step3***: For the remaining individuals, calculate their crowding distance. Compute the distance between each individual and their closest neighbors on each objective, i.e., the difference between the objective values of* x*_*i*+*1*_ and *x*_*i-1*_, and normalize it. The calculation formula is shown in Eq. (19):
  19 $$d_{k} (x_{i} ) = \frac{{\left| {f_{k} (x_{i + 1} ) - f_{k} (x_{i - 1} )} \right|}}{{\max (f_{k} ) - \min (f_{k} )}}$$
  where *d*_*k*_*(x*_*i*_*)* represents the crowding distance of the *i*th individual on the *k*th objective, *x*_*i*+*1*_ and *x*_*i−1*_ are the two objective values adjacent to *x*_*i*_, and max(*f*_*k*_) and min(*f*_*k*_) represent the objective values of the first and last individuals on the *k*th objective.
  ***Step4***: Traverse each objective, sum the normalized crowding distances on each objective, and obtain the final crowding distance *D(x*_*i*_*)* for* x*_*i*_ :
  20 $$D(x_{i} ) = \sum\limits_{k = 1}^{m} {d_{k} (x_{i} )}$$
  ***Step5***: Then, reorder the individuals in the Pareto solution set according to the crowding distance and remove the individuals with the lowest rank.
  ***Step6***: Repeat ***Step3*** to ***Step6*** until the number of individuals in the Pareto solution set meets the requirements.

By introducing the multi-objective optimization framework based on non-dominated sorting and crowding distance sorting into the traditional AHA, we have obtained MOAHA, which expands the applicability of AHA. It enables AHA to solve the RIES low-carbon economic scheduling problem and obtain the optimal Pareto front that describes the RIES low-carbon economic scheduling problem. Furthermore, MOAHA can find the best balance between multiple optimization objectives for RIES, thereby improving the overall performance of RIES.

### Population initialization based on chaotic mapping

For traditional AHA, the initial population is highly random. It often far from the optimal solution, leading to a high degree of randomness and instability in the optimization results, which affects the search efficiency of the optimal solution. This impact is even more pronounced when solving complex RIES low-carbon economic operation problems. Therefore, we introduce chaotic mapping to dynamically and uniformly generate the initial population within the search space to improve population diversity and uniform traversal^26^.

Chaotic mapping is an optimization method based on chaotic mapping rules, specifically used to describe the complex chaotic behavior generated by nonlinear systems. This method has a series of remarkable features such as determinism, apparent randomness, high sensitivity to initial values, and non-periodicity. It is precisely because of these unique attributes that chaotic mapping plays an important role in the population initialization stage of meta-heuristic algorithms^27^. The working principle first involves mapping the variables to be optimized to the value range of chaotic variables. Then, optimization is performed through the characteristics of chaotic variables, and finally, the optimized solutions found in the chaotic space are linearly transferred to the actual optimization space.

Specifically, chaotic systems can be divided into two categories: low-dimensional chaos and high-dimensional chaos. High-dimensional chaotic systems stand out for their complex structure, numerous control parameters, and relatively high computational complexity. In contrast, low-dimensional chaotic systems offer simpler structures, fewer control parameters, and more intuitive implementation methods. However, low-dimensional chaotic systems also have some problems, such as the finiteness of chaotic behavior, the discontinuity of chaotic intervals, and the non-uniform data distribution of generated chaotic sequences.

Different chaotic mappings have different properties, and their effects on optimization algorithms also vary significantly. For example, Cubic and Chebyshev mappings are sensitive to initial values, but mapping values are unevenly distributed in the interval [0,1]. Logistic mapping has strong spatial traversal, but there are blank areas and aggregation areas in the system. Sine mapping has the advantages of simple structure and high efficiency, but there is a problem of uneven probability density distribution. Sinusoidal mapping has a nonlinear feedback mechanism and sensitivity to initial values, but there are blank areas and fixed-point problems. Tent mapping has good correlation and uniform probability density distribution, but it easily decays into periodic sequences in the later stages of iteration. The common types of chaotic mappings are shown in Table 1.

**Table 1:** Introduction to several common chaos mappings.

Mapping type	Formula	Search range
Cubic	$x_{n + 1} = \rho \times x_{n} \times (1 - x_{n}^{2} )$	[0, 1]
Chebyshev	$x_{n + 1} = \cos ({\text{a}} \times {\text{cos}}^{ - 1} (x_{n} )),a = 0.5$	[-1, 1]
Logistic	$x_{n + 1} = \lambda \times x_{n} \times (1 - x_{n} ),\lambda \in (0,4)$	[0, 1]
Sine	$x_{n + 1} = a \times \sin (\pi x_{n} ),a \in [0,1]$	[0, 1]
Sinusoidal	$x_{n + 1} = a \times x_{n}^{2} \times \sin (\pi x_{n} ),a \in [0,4]$	[0, 1]
Tent	$$x_{n + 1} = \left\{ {\begin{array}{*{20}c} {x_{n} /a \, ,x_{n} < a} \\ {(1 - x_{n} )/(1 - a),x_{n} \ge a} \\ \end{array} } \right.$$	[0, 1]

**Figure 4:**
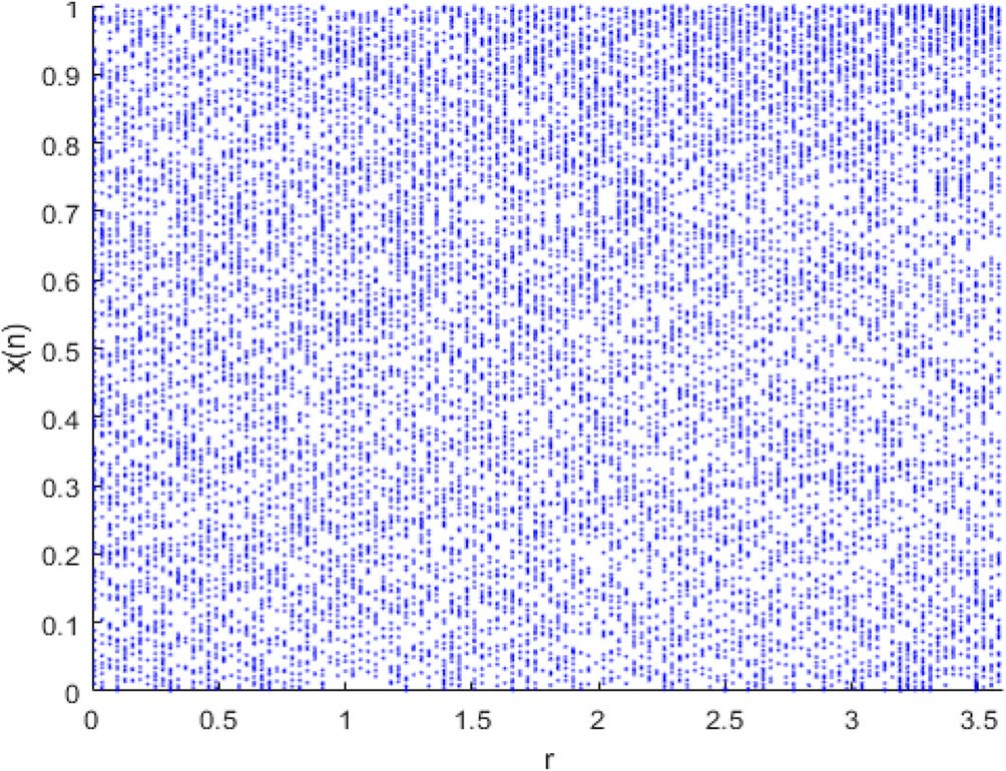
The Logistic-Tent chaos mapping.

Therefore, considering the limitations of ordinary chaos mappings, we chose to combine the Logistic chaos mapping and the Tent chaos mapping to form the Logistic-Tent chaos mapping and use it as the food source initialization method for AHA to enhance algorithm performance and improve the efficiency of solving the regional integrated energy optimization scheduling problem. Figure 4 shows the distribution of the Logistic-Tent chaos mapping, and its formula is shown in Eq. (21):

21 $$x_{n + 1} = \left\{ {\begin{array}{*{20}c} { \, \left[ {\begin{array}{*{20}c} {rx_{n} (1 - x_{n} ) + \frac{(4 - r)}{2}x_{n} } \\ \end{array} } \right]{\text{mod }}1 \, ,{\text{if }}x_{n} < 0.5} \\ {\left[ {\begin{array}{*{20}c} {rx_{n} (1 - x_{n} ) + \frac{{(4 - r)(1 - x_{n} )}}{2}} \\ \end{array} } \right]{\text{mod }}1 \, ,{\text{if }}x_{n} \ge 0.5} \\ \end{array} } \right.$$

where the initial value of *x*_*n*_ is in the range (0,1); the control parameter *r* is in the range (0,4); and mod is the modulus operation.

#### Dynamic adjustment factor

On the other hand, the traditional AHA relies on random numbers to select foraging methods, which results in slower search speed and unstable search capability in the optimization space, affecting the efficiency of searching for scheduling solutions. To enhance both the global and local search capabilities of AHA and improve the algorithm's optimization speed, we introduce a probability dynamic adjustment factor *P*. It adjusts the selection probability of guided foraging or regional foraging based on the dominance level of the scheduling solution, encouraging birds with lower fitness in the population to choose guided foraging with higher probability, thus enhancing global search capability. It also encourages birds with higher fitness in the population to choose regional foraging with higher probability, enhancing local search capability. The probability dynamic adjustment function *P* is represented as:

22 $$P_{i}^{t + 1} = \left\{ {\begin{array}{*{20}c} {P_{\min } + \left( {P_{\max } - P_{\min } } \right)\frac{{F\left( {X_{i}^{t} } \right) - F_{\min }^{t} }}{{F_{mid}^{t} - F_{\min }^{t} }} \, , \, F\left( {X_{i}^{t} } \right) \le F_{mid}^{t} } \\ {1 - P_{\min } - \left( {P_{\max } - P_{\min } } \right)\frac{{F_{\max }^{t} - F\left( {X_{i}^{t} } \right)}}{{F_{\max }^{t} - F_{mid}^{t} }} \, , \, F\left( {X_{i}^{t} } \right) > F_{mid}^{t} } \\ \end{array} } \right.$$

where *P*_*i*_^*t*+*1*^ is the probability dynamic adjustment function of the *i*th bird in the *(t* + *1)*th iteration, *P*_*max*_ and *P*_*min*_ are the preset maximum and minimum probabilities. *F(X*_*i*_^*t*^*)* is the dominance level of the *i*th bird in the *t*th iteration, *F*^*t*^_*max*_ and *F*_*min*_^*t*^ are the highest and lowest dominance levels in the *t*th iteration, and *F*^*t*^_*mid*_ is the median dominance level in the *t*th iteration.

We control the probability of guided foraging and regional foraging using the probability dynamic adjustment factor. When *P*_*i*_^*t*+*1*^ ≤ *P*_*rand*_^*t*^ guided foraging is performed; otherwise, regional foraging is carried out, where *P*_*rand*_^*t*^ is a random number within the range of 0 to 1.

In summary, the proposed MOCAHA can solve multi-objective optimization problems. We have made improvements to address issues with the traditional AHA, such as high randomness in the initial population, slow optimization speed, and unstable search capabilities. These improvements make the multi-objective chaotic artificial hummingbird algorithm suitable for solving the low-carbon economic dispatch problem in RIES.

### Scheduling decision method based on TOPSIS with subjective and objective combined weights

Upon deriving the Pareto front, which delineates the optimal solution set for the multi-objective optimization scheduling of the RIES using MOCAHA, the system's real-time operational prerequisites must be considered to discern a compromise solution. The TOPSIS is widely used in multi-objective optimization decision-making. It can fully utilize the original data information and accurately reflect the gap between various decision-making schemes. The TOPSIS takes the results in the optimal solution set as decision-making schemes, calculates the relative closeness to the ideal solution, and takes the solution with smaller relative closeness as the effective compromise solution. Nonetheless, conventional TOPSIS harbors two principal shortcomings: firstly, the metric's weight is predominantly dictated by expert opinion, introducing an element of subjectivity. Secondly, its reliance on Euclidean distance for computing the disparity between each proposal and the archetype tends to obscure the intrinsic merits and drawbacks of individual plans. Specifically, when multiple metrics are at play, a scheme proximate to the positive ideal solution might, paradoxically, also be near its negative counterpart when viewed through the lens of Euclidean distance.

We proposed a TOPSIS based on subjective and objective combined weights. After obtained the Pareto optimal solution set, we use the entropy weight TOPSIS that considers subjective weight correction. First, the optimal compromise solution is selected from the solution set to construct an evaluation model. A comprehensive evaluation is performed based on the entropy weight method, and the objective weight is sought, considering the impact of the Pareto optimal solution difference. Then, combine the subjective weight determined by the dispatching expert experience to make appropriate corrections to the objective weight and obtain the subjective and objective combined weights. The method used considers the entropy weight and subjective weight of the objective function comprehensively when calculating the relative closeness. It takes into account the experience of scheduling decision-making experts while reflecting the importance of the two objective functions objectively. According to this, the Pareto optimal solution with the maximum relative closeness value is selected as the optimal compromise solution. Based on the data of N optimal points on the Pareto front, a model is established and a comprehensive evaluation is conducted. The specific steps are as follows:

***Step1***: Establish an evaluation matrix.

For the two objective functions we established and the N Pareto optimal solutions, an evaluation matrix $R^{\prime}$ is established:

23 $$R^{\prime} = \left[ {\begin{array}{*{20}c} {r_{11}^{\prime} } & {r_{12}^{\prime} } & {...} & {r_{1N}^{\prime} } \\ {r_{21}^{\prime} } & {r_{22}^{\prime} } & {...} & {r_{2N}^{\prime} } \\ \end{array} } \right]$$

When *i* takes values of 1 and 2, *rij’* is the value of the *i*th objective function corresponding to the *j*th Pareto optimal solution.

***Step2***: Normalization of data.

Since there are differences in dimensions and orders of magnitude between objective functions, the original data can be normalized as follows:

24 $$r_{ij} = \frac{{\max (r_{ij}^{\prime} ) - r_{ij}^{\prime} }}{{\max (r_{ij}^{\prime} ) - \min (r_{ij}^{\prime} )}}$$

where *r*_*ij*_ is the normalized value of the *i*th objective function corresponding to the *j*th Pareto optimal solution; and $\max (r_{ij}^{\prime} )$ and $\min (r_{ij}^{\prime} )$ are the maximum and minimum values of the *i*th row in *R*, respectively. The normalized evaluation matrix *R* is:

25 $$R = \left[ {\begin{array}{*{20}c} {r_{11} } & {r_{12} } & {...} & {r_{1N} } \\ {r_{21} } & {r_{22} } & {...} & {r_{2N} } \\ \end{array} } \right]$$

***Step3***: Using entropy weight method to calculate the objective weight of each objective function.

First, calculate the entropy of each objective function by Eq. (26):

26 $$e_{i} = - \frac{{\sum\nolimits_{j = 1}^{N} {\left[ {\frac{{r_{ij} }}{{\sum\nolimits_{j = 1}^{N} {r_{ij} } }} \times \ln \left( {\frac{{r_{ij} }}{{\sum\nolimits_{j = 1}^{N} {r_{ij} } }}} \right)} \right]} }}{\ln N},\quad i = 1,2$$

where *e*_*i*_ is the entropy of the *i*-th objective.

The entropy weights is determined by the degree of difference in the solutions under this objective, which represents the amount of information provided by this objective. The formula for calculating the entropy weight is:

27 $$\alpha_{i} = \frac{{1 - e_{i} }}{{\sum\nolimits_{j = 1}^{2} {(1 - e_{j} )} }},\quad i = 1,2$$

where *α*_*i*_ is the entropy weight of the *i*-th objective.

***Step4***: Use the expert weights of dispatching experience to obtain the subjective and objective combined weights, the equation is:

28 $$\omega_{i} = \frac{{\alpha_{i} \lambda_{i} }}{{\sum\nolimits_{i = 1}^{2} {\alpha_{i} \lambda_{i} } }}$$

where *λ*_*i*_ is the subjective weight set by the scheduling expert, and *ω*_*i*_ is the subjective and objective combined weights.

As you can see, *ω*_*i*_ considers both the work experience of dispatchers and the entropy weights that objectively reflects the degree of difference between different solutions on the Pareto front.

***Step5***: Establish a weighted normalized evaluation matrix $\hat{R}$

29 $$\hat{R} = \left[ {\begin{array}{*{20}c} {\omega_{1} r_{11} } & {\omega_{1} r_{12} } & {...} & {\omega_{1} r_{1N} } \\ {\omega_{2} r_{21} } & {\omega_{2} r_{22} } & {...} & {\omega_{2} r_{2N} } \\ \end{array} } \right]$$

In the matrix $\hat{R}$, the maximum and minimum values of the *i*th row correspond to the most ideal and least ideal situations for the *i*th objective, respectively.

***Step6***: Determine the positive and negative ideal points.

The formulas for calculating the positive ideal point *F*^+^ and the negative ideal point *F*^*-*^ are followed:

30 $$\left\{ \begin{gathered} f_{i}^{ + } = \min (\hat{R}_{iN} ,\hat{R}_{iN} ,...,\hat{R}_{iN} ),\quad i = {1,2} \hfill \\ F^{ + } = (f_{1}^{ + } ,f_{2}^{ + } ) \hfill \\ \end{gathered} \right.$$

31 $$\left\{ \begin{gathered} f_{i}^{ - } = \max (\hat{R}_{iN} ,\hat{R}_{iN} ,...,\hat{R}_{iN} ),\quad i = {1,2} \hfill \\ F^{ - } = (f_{1}^{ - } ,f_{2}^{ - } ) \hfill \\ \end{gathered} \right.$$

where $\hat{R}_{iN}$ is the value of *i*th row and *N*th column in the matrix $\hat{R}$; min(·), max(·) indicate the minimum and maximum values, respectively.

**Step7**: Calculate the relative closeness of each Pareto optimal solution

32 $$T_{j} = \frac{{D_{j}^{ - } }}{{D_{j}^{ + } + D_{j}^{ - } }}$$

where *T*_*j*_ is the relative closeness of the *j*th solution. *D*_*j*_^+^ and *D*_*j*_^-^ are the Euclidean distances from the *j*th solution to the positive ideal point and the negative ideal point, respectively.

The higher the relative closeness value, the closer the solution is to the positive ideal point. Therefore, the Pareto optimal solution with the highest relative closeness is selected as the optimal compromise solution.

In summary, to address the inherent challenges in RIES low-carbon economic dispatch, such as susceptibility to local optima, delayed solution speed, and unpredictable outcomes, we've advanced a RIES low-carbon economic dispatch method based on MOCAHA. Firstly, this method employs the multi-objective chaotic artificial hummingbird algorithm to solve the RIES low-carbon economic problem, obtaining a uniformly distributed Pareto optimal frontier, thereby optimizing both the economic and low-carbon operation of RIES. Then, based on the proposed TOPSIS-SOCW and by integrating the objective features of the Pareto solution set with the expert judgment of dispatchers, an optimal dispatch scheme that balances the environmental protection and economic aspects of RIES is obtained. In essence, our approach not only provides a solution to the low-carbon economic scheduling challenges of RIES but also fortifies the system's energy efficiency, achieving a tangible low-carbon economic operation for RIES.

## Case study

### Introduction to the simulation case

This paper investigates the RIES located in the northeastern region of China primarily. The climate characteristics of this region are hot summers and cold winters, with a particularly prominent demand for thermal energy. Additionally, the northeastern region possesses a developed natural gas pipeline system and abundant wind power resources, offering immense potential for the development of RIES.

**Figure 5:**
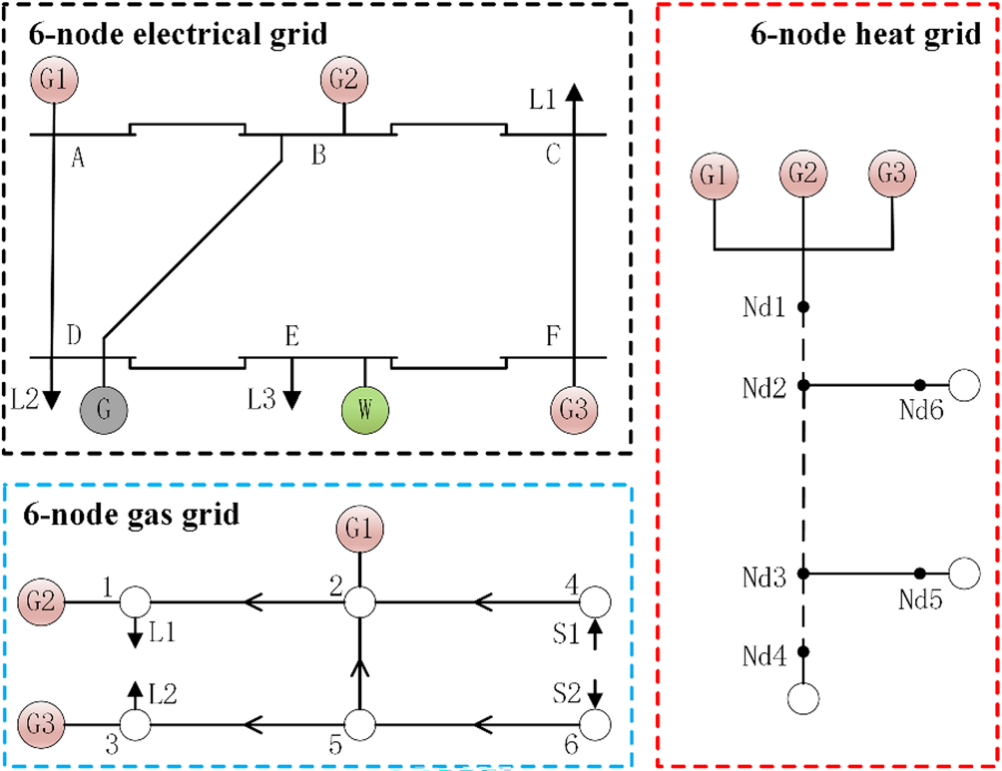
Topology of the simulation example.

To enhance the energy efficiency of the Northeast's regional integrated energy system holistically, we adopted a comprehensive simulation case. This comprises a cutting-edge IEEE 6-node electric grid, coupled with a 6-node gas grid and a 6-node thermal grid^28,29^. Figure 5 delineates the simulation system's topology. This simulation case considers the coupling relationships among the electric power system, thermal system, and natural gas system adequately. It also introduces a significant amount of wind power resources to simulate the actual energy supply and demand characteristics of the northeastern region. By leveraging this simulation case, we aspire to probe deeper into the operational nuances and optimization strategies of RIES specific to the Northeast. In doing so, we aim to offer robust theoretical insights that champion efficient energy consumption and further the region's sustainable progress.

**Figure 6:**
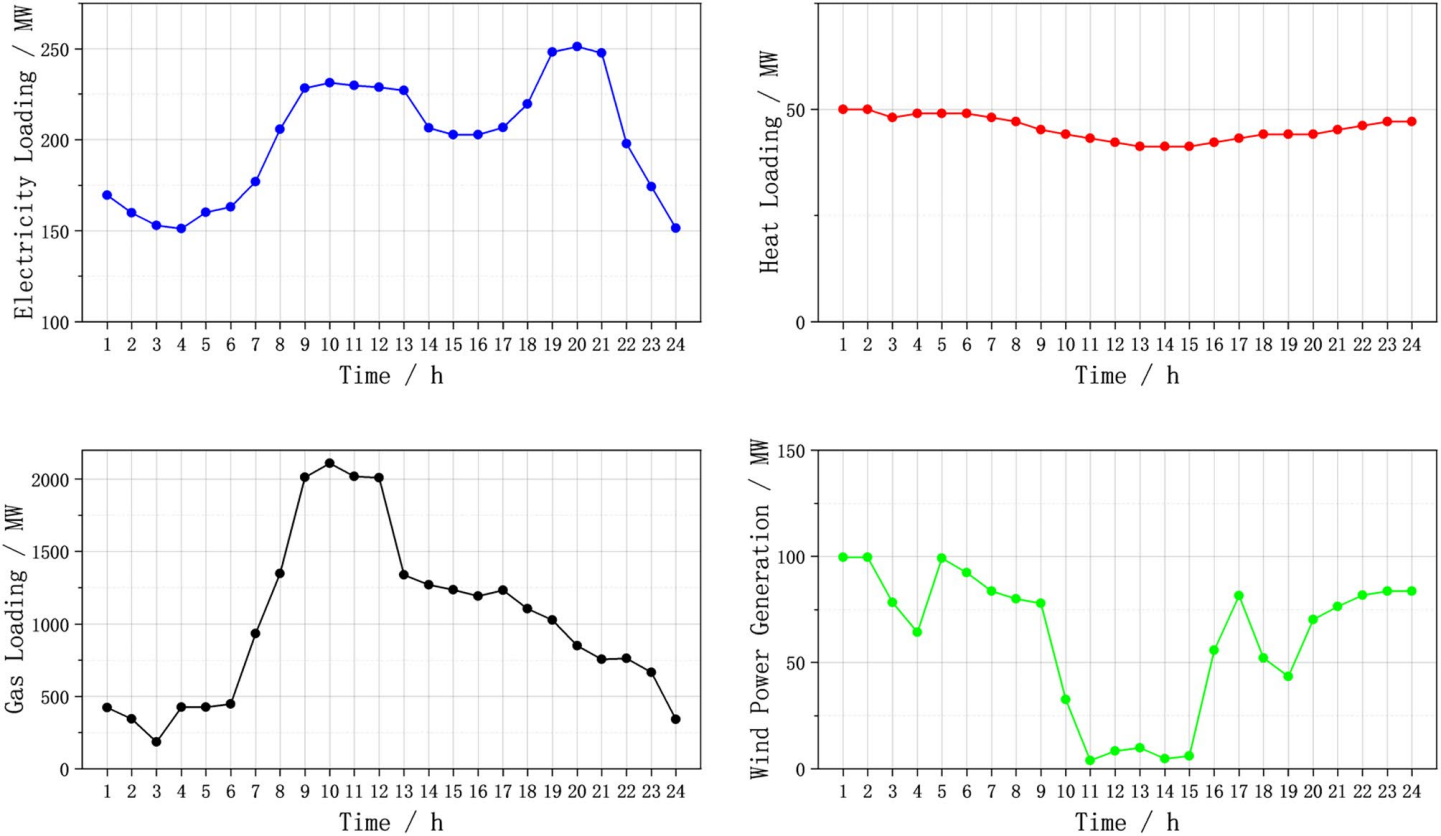
Predicted data of electric, thermal, gas load, and wind turbine output.

In this case, the electrical grid, heat network, and gas network are interconnected through three CHP units that run on gas. Additionally, the electrical grid incorporates one coal-fired power unit and one wind power unit. The parameters of the units used in this case are detailed in Table 2. The load forecast curve, wind power plant output forecast curve and other parameters are shown in Fig. 6. The time-of-use purchase price of electricity from the main grid for users is shown in Table 3. The gas source purchase price is 1.8$/kcf, and the heat source purchase price is 20$/MW·h. The penalty price for curtailment of wind power is 50$/MW·h. The penalty prices for reducing electric load and gas load are 100$/MW h and 5$/kcf, respectively. Other system parameters can be found in the Refs^30,31^.

**Table 2:** Unit parameters.

ID	G1	G2	G3	G4
Type	CHP	CHP	CHP	Thermal generator
P_min_/MW	30	10	10	10
P_max_/MW	100	80	80	50
R_amp_/(MW/h)	50	40	40	20
a/($/h)	–			137.41
b/($/MWh)	–			37.7
c/($/MWh)	–			0.005
Startup cost/gas consumption	500kcf			500$
Other	k_CHP_ = 2.62, n_CHP_ = 0.6			–

**Table 3:** Time-of-use electricity prices.

Time	Price ($/MW)	Time	Price ($/MW)	Time	Price ($/MW)	Time	Price ($/MW)
1	18	7	23	13	23	19	18
2	18	8	23	14	23	20	18
3	18	9	23	15	23	21	18
4	18	10	21	16	23	22	18
5	18	11	21	17	21	23	18
6	18	12	21	18	21	24	18

We focus on the day-ahead scheduling of the system, with a scheduling period of 24 h and a scheduling time step set at 1 h. The simulation experiments are conducted on a computer with a 2.40 GHz, 16 GB RAM, AMD Ryzen R7-5800H CPU, running Windows 11, and the experiments are carried out on the MATLAB R2020b software platform.

### Comparison of multi-objective optimization algorithm performance

In this paper, we benchmarked our proposed multi-objective chaotic artificial hummingbird algorithm (MOCAHA) against four renowned algorithms: the multi-objective artificial hummingbird algorithm (MOAHA), the multi-objective multi-verse optimization (MOMVO) algorithm^13^, the multi-objective gray wolf optimization (MOGWO) algorithm^28^, and the multi-objective particle swarm optimization (MOPSO) algorithm^29^. Each algorithm was tasked with solving the case study model outlined in this paper. To maintain consistency in the evaluation, we standardized the iteration times^3,32,33^ for all algorithms to 1000, set the population size at 100, and kept the external archive size at 50.

Table 4 presents the statistical results of the Pareto frontier obtained by the five algorithms after solving the regional integrated energy system's low-carbon economic operation problem. According to the table, compared to MOAHA, MOCAHA can enhance the optimization effect of economic cost while ensuring the optimization effect of carbon emissions. It improves the reduction of economic costs by an average of 8.87% compared to MOAHA; compared to MOMVO, MOCAHA has an advantage in reducing carbon emissions, with an average improvement of 5.55%; compared to MOGWO, MOCAHA has a significant advantage in reducing economic costs, with an average improvement of 30.62%; compared to MOPSO, MOCAHA has significant advantages in reducing both economic costs and carbon emissions, with average improvements of 26.60% and 11.13% respectively. In addition, among the five algorithms, MOCAHA has the largest difference between its best and worst solutions, indicating a broader Pareto solution set distribution and stronger global search capability. Simultaneously, MOCAHA has the smallest best solutions in both reducing economic costs and carbon emissions, indicating the strongest local search capability among the algorithms. Therefore, MOCAHA can obtain a more reasonable set of Pareto non-dominated solutions, providing decision-makers with multiple candidate plans with different preferences.

**Table 4:** Pareto optimization results obtained by different algorithms.

Algorithm	Index	Cost/$	Carbon emissions/t
MOCAHA	Min	115,122.99	16,420.02
	Average	119,564.11	17,128.63
	Max	127,933.66	17,844.13
MOAHA	Min	122,303.17	16,567.65
	Average	126,322.76	17,158.62
	Max	132,053.85	17,801.79
MOMVO	Min	117,686.03	17,944.55
	Average	118,365.19	18,135.40
	Max	120,256.70	18,252.69
MOGWO	Min	169,396.35	17,165.04
	Average	172,326.36	17,511.06
	Max	177,991.20	18,038.22
MOPSO	Min	160,519.91	19,171.24
	Average	162,902.63	19,273.01
	Max	165,780.96	19,471.67

**Figure 7:**
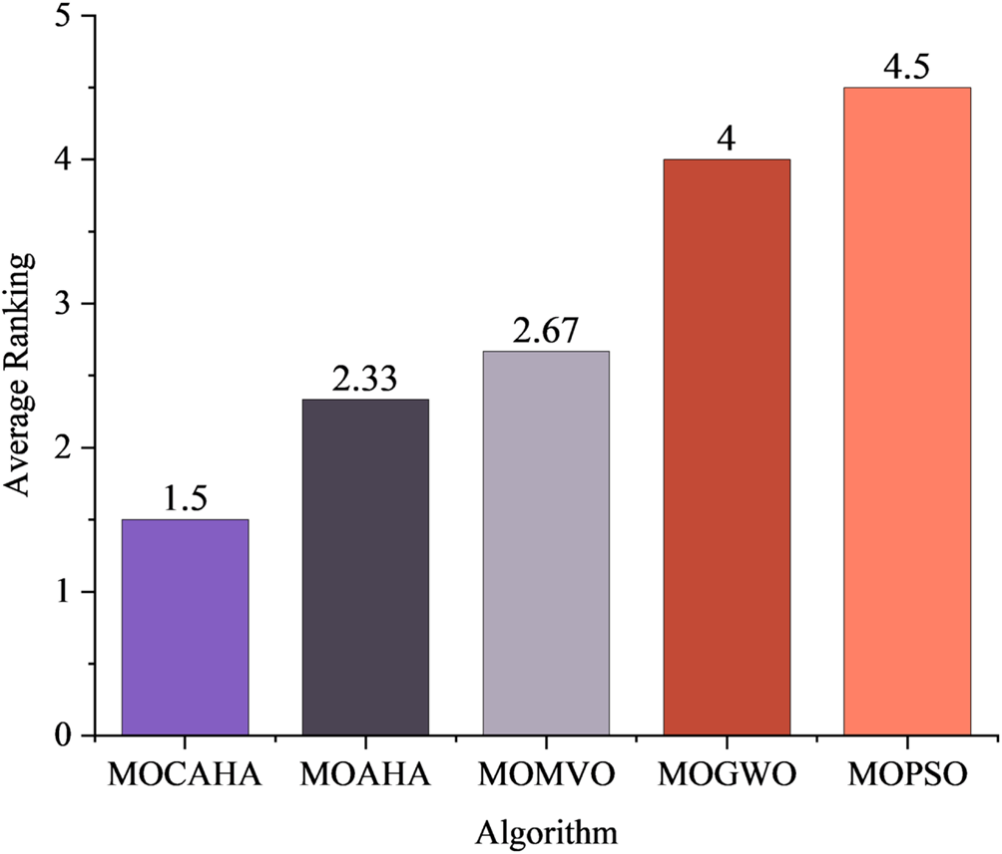
Average ranking of the optimization performance of each algorithm.

The best, average, and worst values of the five algorithms under each objective are ranked, and the average ranks are taken. The average rankings of each algorithm are shown in Fig. 7. It can be seen that the average rank of MOCAHA is 1.5, ranking first among the five algorithms.

When addressing practical multi-objective optimization problems, the superior performance of a multi-objective optimization algorithm is reflected in both the breadth of the Pareto solution set distribution and its exceptional convergence speed^34^.

**Figure 8:**
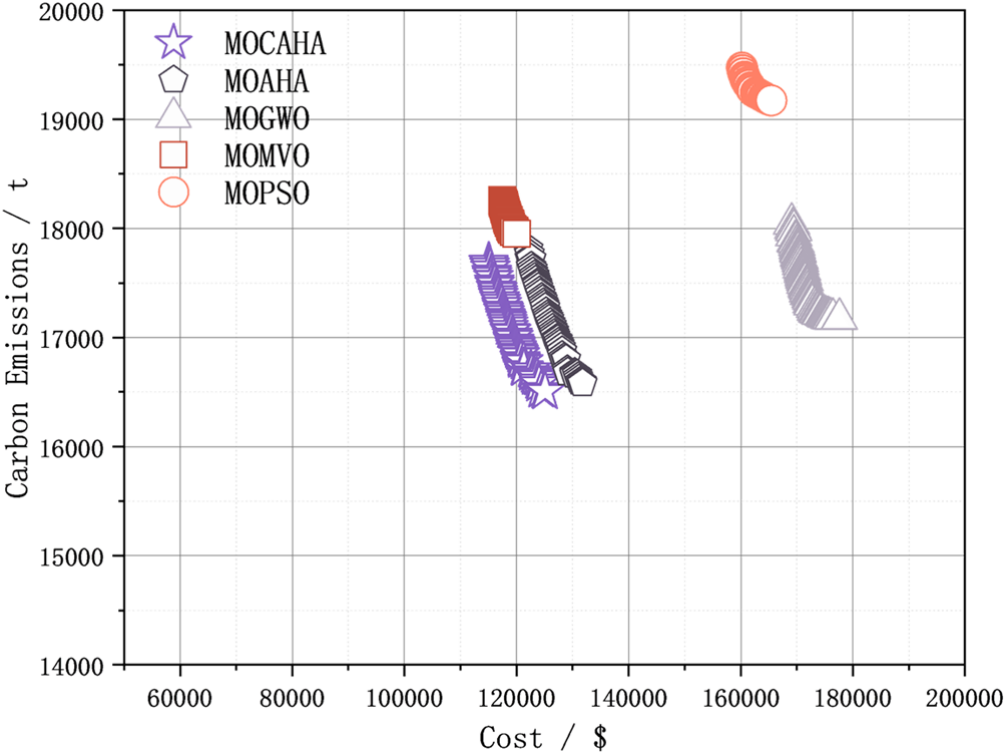
Comparison of the final Pareto frontier obtained by the five algorithms when solving the model.

A widely distributed Pareto solution set implies that the algorithm is more adept at capturing the diversity and complexity within the problem space, providing decision-makers with a more comprehensive set of choices. This is particularly crucial in practical scenarios where the complexity and diversity of problems often involve multiple competitive objectives. Figure 8 shows the comparison of the final Pareto frontiers obtained by the five algorithms in the simulation system of this paper. The Pareto frontier of different algorithms is formed by the solutions in the external archive at the end of their iterations. Analysis shows that MOCAHA, when solving the low-carbon and economic operation problem of the regional integrated energy system, can dominate the solution sets obtained by all other algorithms and has the best optimization effect in terms of economic cost and carbon emissions. Furthermore, as can be seen from Fig. 8, under the same conditions of the number of iterations, population size, and external archive set size, the Pareto frontier obtained by MOCAHA has a wider extension range and more uniform distribution.

**Figure 9:**
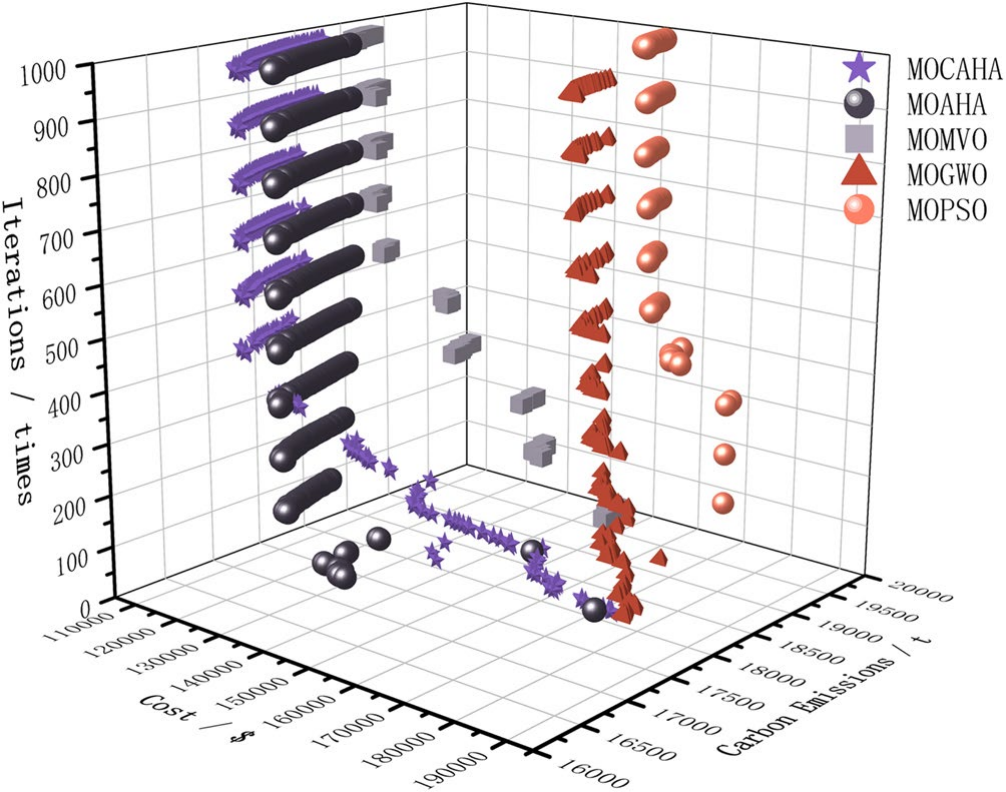
The change process of the external archive during the iterative solving of the model by the five algorithms.

Simultaneously, excellent convergence speed is equally vital for the practical application of multi-objective optimization algorithms^35^. A fast and stable convergence capability enables the algorithm to swiftly identify solution sets that are close to the optimal under limited computational resources, thereby enhancing the algorithm's practicality and efficiency. In dealing with real-world problems, the ability to rapidly obtain high-quality solutions is paramount for the practical use of optimization algorithms, especially in contexts involving complex decision-making and resource constraints. Figure 9 is obtained by saving the population in the Archive set every 100 iterations for each algorithm. Analysis shows that, after introducing the population initialization and dynamic adjustment factors based on the Logistic-Tent chaotic mapping, our proposed MOCAHA has better convergence in the early stage of the algorithm iteration than MOGWO, MOMVO, MOPSO, and ranks second only to MOAHA. However, in the later stage of the iteration, MOCAHA's optimization ability ranks first among the five algorithms and has a clear advantage.

In addition, we ran each of the five algorithms 20 times randomly, combining the objective values of the 100 sets of Pareto non-dominated solutions obtained to determine the Pareto frontier of the combined solution set, which is used as the approximate Pareto optimal frontier for all algorithms to solve this multi-objective optimization problem^36^. Then, we used the convergence, diversity, and comprehensive performance metrics from^37^ to validate the overall performance of the RIES low-carbon economic operation solutions obtained by the five algorithms^38^.

Convergence metrics usually calculate the distance between the solution set obtained by the multi-objective optimization algorithm and the Pareto approximate frontier to reflect the closeness of the solution set to the real Pareto frontier. Different metrics choose different types of distances, and most require a reference set for comparison, i.e., an external reference set or another solution set. The better the convergence of the solution set, the better the convergence of the multi-objective optimization algorithm used. We used Generational Distance (GD) and epsilon (ε) to test and compare the convergence of the algorithms, and the results are shown in Table 5.

**Table 5:** Comparison of convergence metrics.

Metric	MOCAHA	MOAHA	MOMVO	MOGWO	MOPSO
GD					
Min	4.5749E + 00	6.7168E + 00	3.7058E + 01	4.6321E + 03	2.4571E + 03
Average	2.5590E + 01	4.9291E + 01	5.6994E + 01	6.0025E + 03	2.5997E + 04
Max	9.7401E + 01	1.9168E + 02	8.3846E + 01	7.1049E + 03	1.1492E + 05
ε					
Min	1.6254E + 02	2.8649E + 02	2.1511E + 03	5.3934E + 04	3.4710E + 04
Average	1.1322E + 03	1.1406E + 03	4.3274E + 03	6.6648E + 04	2.0391E + 05
Max	3.4265E + 03	2.4147E + 03	6.0738E + 03	7.8544E + 04	8.3199E + 05

According to Table 5, MOCAHA has the best performance in both GD and ε indicators for both the best and average performance, ranking first among the five algorithms. The maximum value of GD is only higher than that of MOMVO, and the maximum value of ε is only higher than that of MOAHA, both ranking second. In summary, among the five algorithms, MOCAHA has the best convergence performance in solving the low-carbon economic operation problem of regional integrated energy systems.

Diversity metrics measure the distribution and extent of the solution set obtained by the multi-objective optimization algorithm. The more uniformly the solution set is distributed, the better its distribution; the denser the solution set is distributed in the boundary area of the Pareto frontier, the better its extent. We use Δ, Coverage over the Pareto Front (CPF), and Maximum Spread (MS) to test and compare the convergence of the algorithms. Where, the smaller the Δ means the better the diversity of the solution set. The larger the CPF and MS, the better the diversity of the solution set. The results are shown in Table 6.

**Table 6:** Comparison of diversity metrics.

Metric	MOCAHA	MOAHA	MOMVO	MOGWO	MOPSO
Δ					
Min	4.8799E − 01	5.2535E − 01	9.2840E − 01	9.7618E − 01	1.0001E + 00
Average	7.1845E − 01	8.0070E − 01	9.6707E − 01	9.8802E − 01	1.0033E + 00
Max	1.1270E + 00	1.1146E + 00	1.0183E + 00	1.0013E + 00	1.0128E + 00
CPF					
Min	3.5001E − 02	5.9497E − 02	0.0000E + 00	0.0000E + 00	0.0000E + 00
Average	2.2805E − 01	1.5537E − 01	9.7332E − 03	0.0000E + 00	0.0000E + 00
Max	6.4372E − 01	2.8011E − 01	4.0000E − 02	0.0000E + 00	0.0000E + 00
MS					
Min	6.8679E − 01	6.7781E − 01	1.8653E − 01	2.3166E − 01	8.1415E − 02
Average	9.8587E − 01	9.0638E − 01	3.3467E − 01	9.1825E − 01	2.7864E − 01
Max	1.3429E + 00	1.3277E + 00	5.0232E − 01	1.3825E + 00	4.5913E − 01

According to Table 6, the average performance of the solution set obtained by MOCAHA is the best in both the Δ and CPF. In the MS, the average performance of MOCAHA is slightly worse than MOGWO, comparable to MOAHA, but the best performance ranks first among the five algorithms. Therefore, the solution set obtained by MOCAHA has good distribution and extent and can meet the diversified needs of the solution set.

The comprehensive metric can simultaneously measure the convergence and diversity of the solution set. Suppose there are two solution sets, S1 and S2. If S1 is better than S2 in the value of a certain comprehensive metric, it means that S2 is better than S2 in convergence or diversity, and it is possible to be better than the solution set S2 in both performances at the same time. We use Hyper Volume (HV), Inverted Generational Distance (IGD), IGDp, and Δp as four indicators to quantitatively evaluate the comprehensive performance of the Pareto solution set of the algorithm. The results are shown in Table 7.

**Table 7:** Comparison of comprehensive performance metrics.

Metric	MOCAHA	MOAHA	MOMVO	MOGWO	MOPSO
HV					
Min	1.9941E − 02	2.0589E − 02	1.4628E − 02	0.0000E + 00	0.0000E + 00
Average	2.2784E − 02	2.2684E − 02	1.6003E − 02	0.0000E + 00	0.0000E + 00
Max	2.4080E − 02	2.3699E − 02	1.7391E − 02	0.0000E + 00	0.0000E + 00
IGD					
Min	6.8469E + 01	1.0286E + 02	9.4621E + 02	4.9680E + 04	3.0493E + 04
Average	2.0389E + 02	2.1610E + 02	1.6308E + 03	6.2393E + 04	2.6426E + 05
Max	8.8656E + 02	5.1538E + 02	2.5433E + 03	7.4286E + 04	1.1526E + 06
IGDp					
Min	2.8295E + 01	6.3605E + 01	6.7305E + 02	4.9680E + 04	3.0493E + 04
Average	1.6972E + 02	1.8688E + 02	1.5080E + 03	6.2393E + 04	2.6426E + 05
Max	8.8524E + 02	5.0785E + 02	2.5135E + 03	7.4286E + 04	1.1526E + 06
Δp					
Min	6.8469E + 01	1.1071E + 02	9.4621E + 02	4.9680E + 04	3.0493E + 04
Average	2.1258E + 02	2.9234E + 02	1.6308E + 03	6.2393E + 04	2.6426E + 05
Max	8.8656E + 02	8.0998E + 02	2.5433E + 03	7.4286E + 04	1.1526E + 06

According to Table 7, the average performance of the solution set obtained by MOCAHA is the best in all three evaluation indicators among the five algorithms, and the worst performance is worse than MOAHA slightly. Therefore, it can be seen that MOCAHA has excellent performance on all comprehensive indicators.

**Figure 10:**
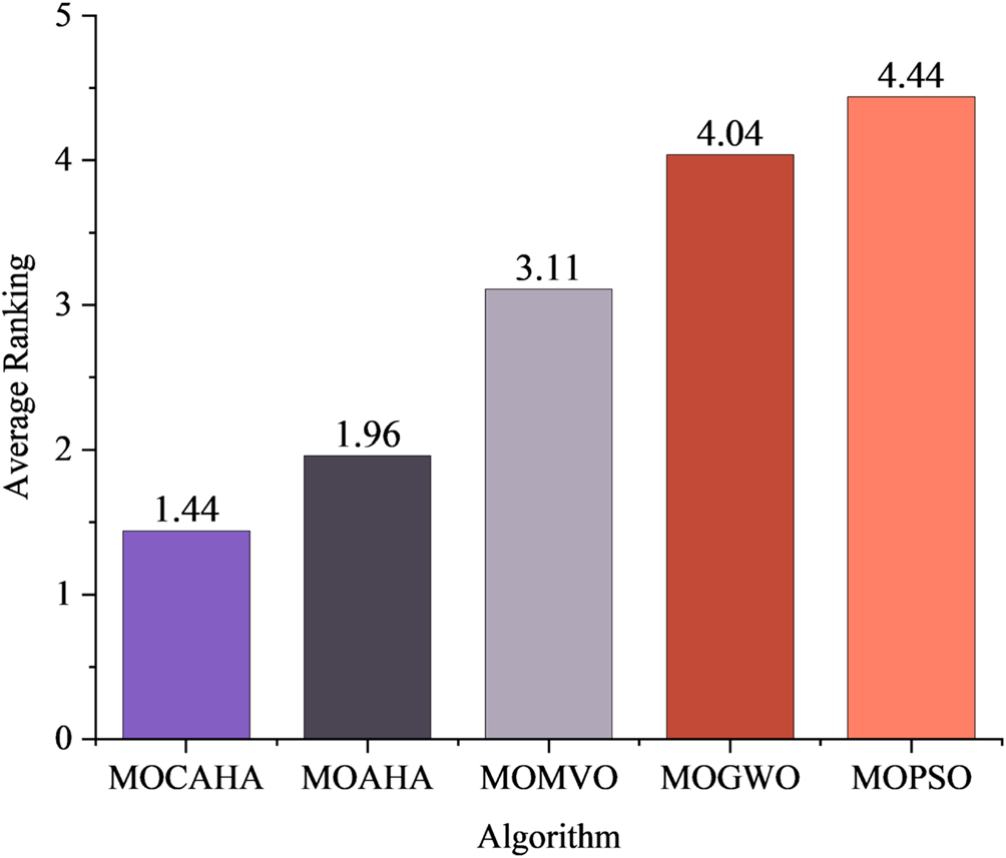
Average ranking of algorithm indicators.

The average rankings of each performance metric for each algorithm were calculated, resulting in an average ranking of the five algorithms for solving RIES multi-objective optimization operation problems. The results are shown in Fig. 10. It can be seen that the average ranking of MOCAHA is 2.31, which is significantly higher than MOAHA, MOMVO, MOGWO, and MOPSO. In summary, MOCAHA has the best overall performance in handling RIES operation optimization problems and possesses excellent search capability for the optimal solution and Pareto solution set optimization.

### Comparison of low-carbon economic dispatch methods performance

To assess the efficacy of the RIES low-carbon economic dispatch approach introduced in this paper, particularly its impact on decreasing operational economic costs and carbon emissions, we employed several methods for comparison. These included our proposed RIES dispatch method utilizing MOCAHA, alongside methods leveraging AHA, MOMVO, and MOPSO. We then conducted a thorough comparative analysis of the obtained operational outcomes. For all strategies that incorporated subjective weights, we standardized the subjective weight vectors to a default value of [0.5, 0.5].

**Figure 11:**
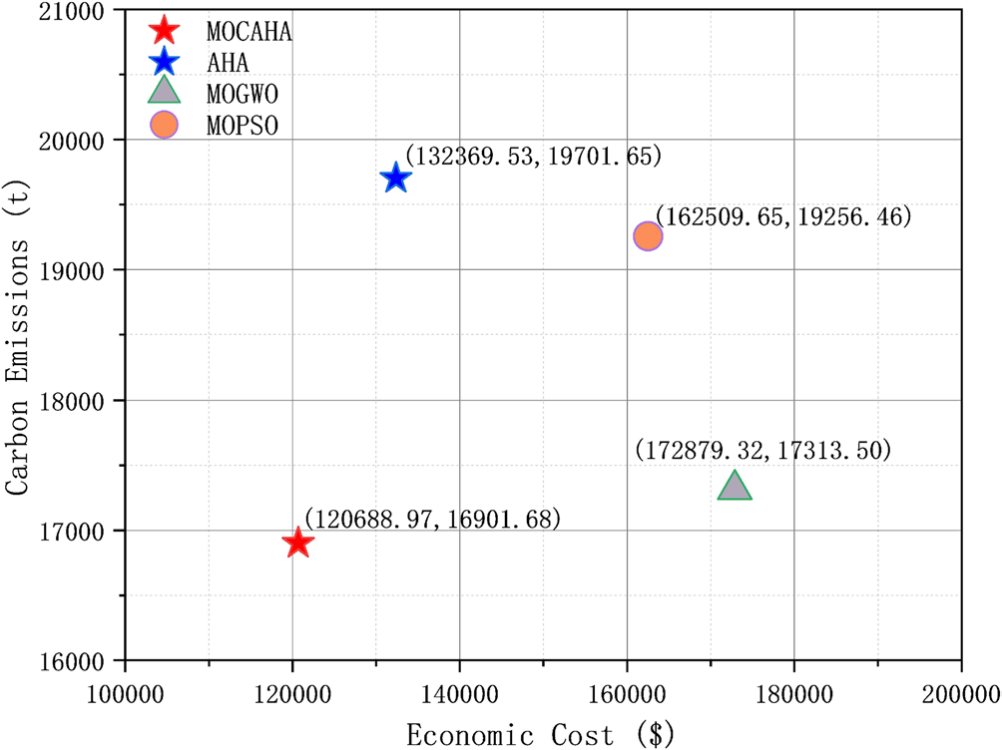
Economic costs and carbon emissions generated by system operation under different solving methods.

The final solutions obtained by each method and their corresponding system operating economic costs and carbon emissions are shown in Table 8 and Fig. 11.

**Table 8:** Economic costs and carbon emissions generated by system operation under different methods.

Method	Cost/$	Carbon emissions (t)
MOCAHA	120,688.97	16,901.68
AHA	132,369.53	19,701.65
MOGWO	172,879.32	17,313.50
MOPSO	162,509.65	19,256.46

**Figure 12:**
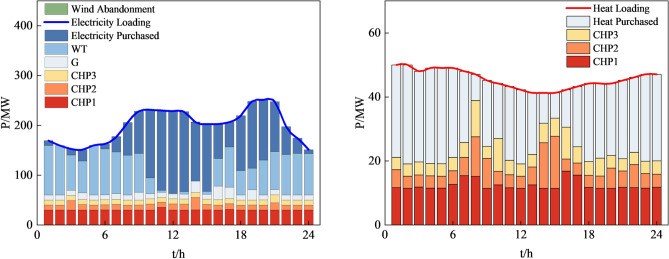
System operation plan obtained by the method based MOCAHA.

**Figure 13:**
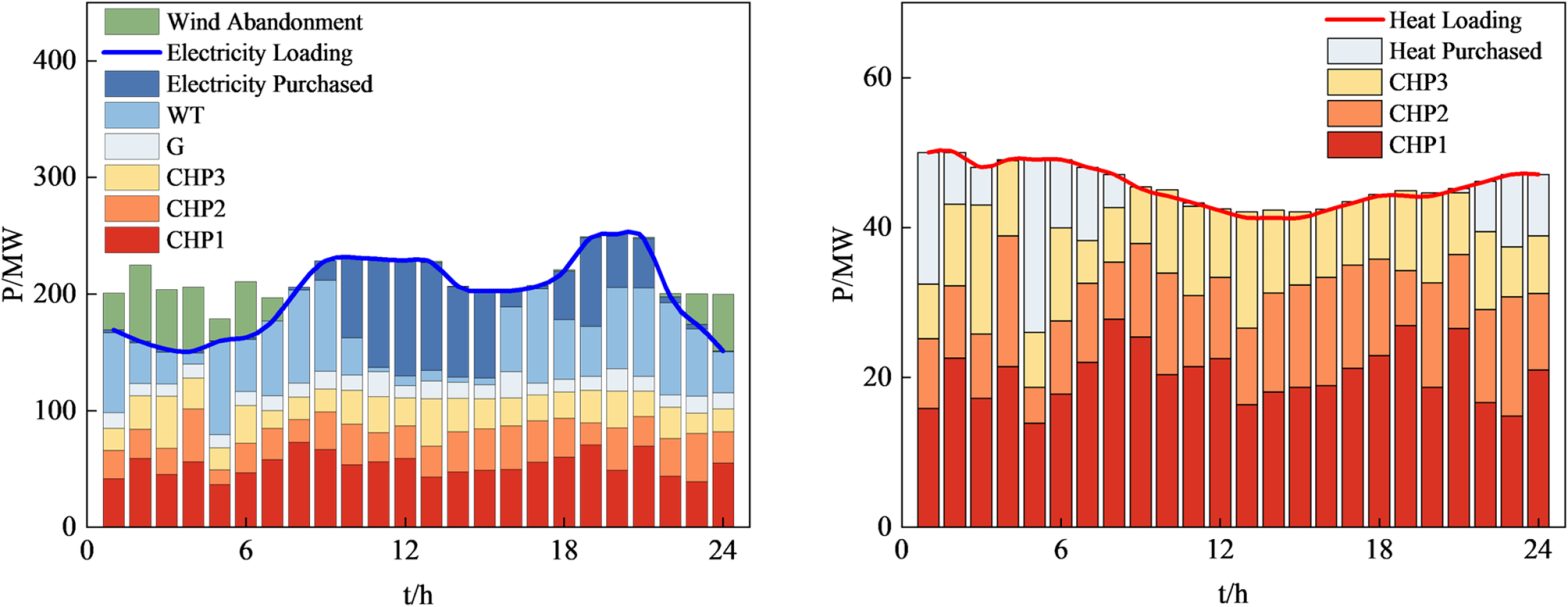
System operation plan obtained by the method based on AHA.

**Figure 14:**
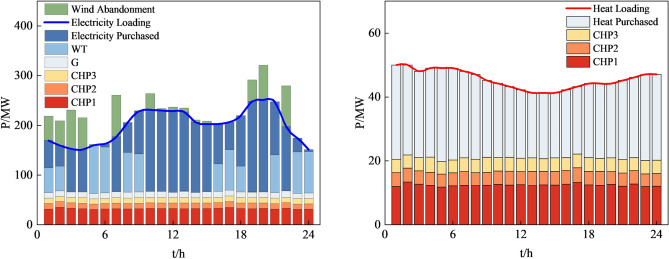
System operation plan obtained by the method based on MOGWO.

**Figure 15:**
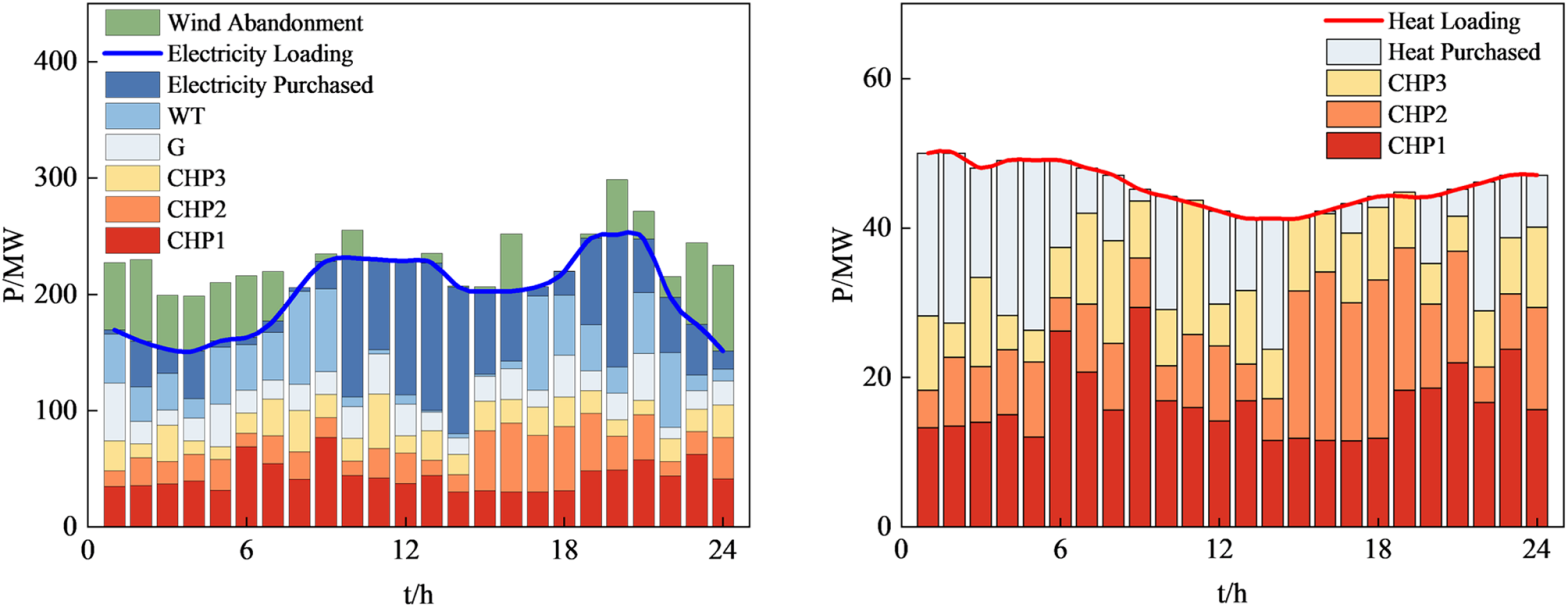
System operation plan obtained by the method based on MOPSO.

Figures 12, 13, 14 and 15, along with Fig. 11 and Table 8, reveals the optimization results obtained from four different methods. Our proposed approach yields both lower economic cost and carbon emissions compared to the method based on AHA, with reductions of 8.8% and 14.2%, respectively. Compared to the method based on MOGWO, our method demonstrates a significant advantage in terms of economic cost, reducing the system's operational costs by 30.2%, and the carbon emissions decrease by 2.3%. When compared to the method based on MOPSO, our proposed approach results in reductions of 25.7% in economic cost and 12.2% in carbon emissions.

In summary, our proposed approach exhibits significant optimization benefits compared to the other three methods. It reduces the system's operational economic cost and carbon emissions effectively. Furthermore, our method ensures the basic consumption of wind power, substantially lowering the curtailment of renewable energy sources. The simulation results further confirm that the method we proposed is an efficient, feasible, and economically and environmentally beneficial dispatch strategy, contributing to achieving low-carbon economic operation in RIES.

### Impact of different subjective weights on simulation results

Upon deriving the Pareto frontier through MOCAHA, our introduced TOPSIS-SOCW technique can seamlessly blend the objective traits of the Pareto frontier with the subjective insights of scheduling professionals. It's worth noting that, during real-time system operations, these experts have the leverage to mold system outcomes by adjusting subjective weights grounded in their expertise.

To attest the method's adeptness in juxtaposing the solution set's objective facets and dispatch experts' seasoned discernment, we have laid out a triad of solution blueprints, delineated in Table 9. These three schemes have different subjective weight vectors, simulating three scenarios in actual system operation where dispatch experts prioritize system economics, low-carbon economic balance, and environmental protection respectively. Using these three schemes, we solve the case and conduct a comparative analysis of the results to clarify the influence of different subjective weights on the final operation results of RIES.

**Table 9:** Three optimization schemes with different subjective weights.

Scenarios	*λ*	
	Cost	Carbon emission
1	0.9	0.1
2	0.5	0.5
3	0.1	0.9

**Figure 16:**
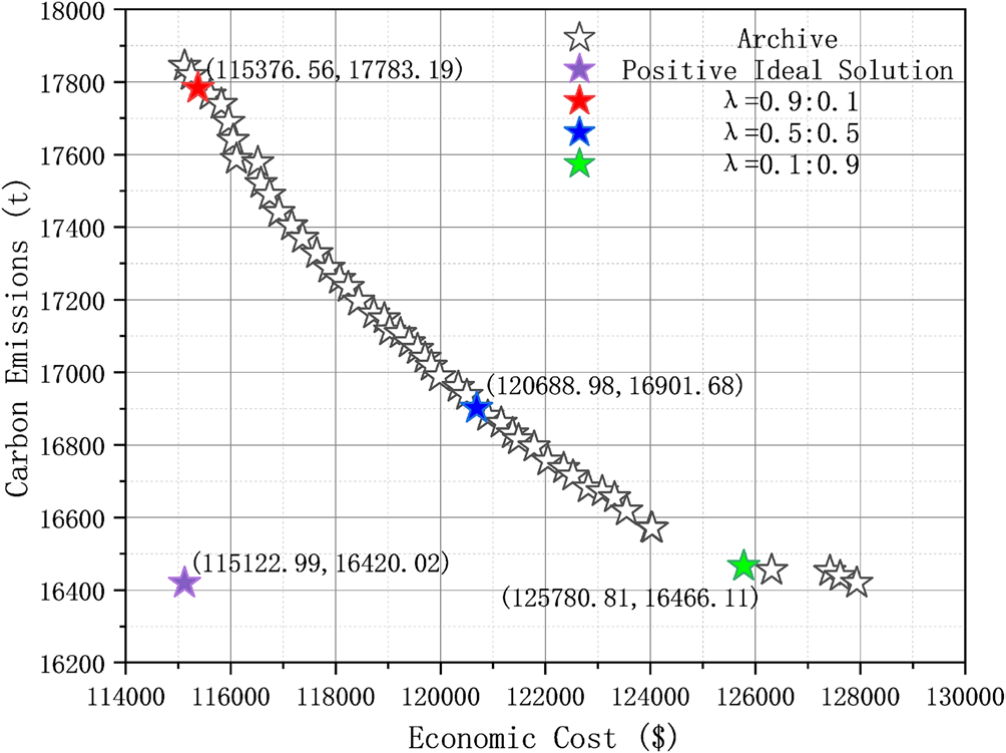
The positions of results on the Pareto frontier.

**Figure 17:**
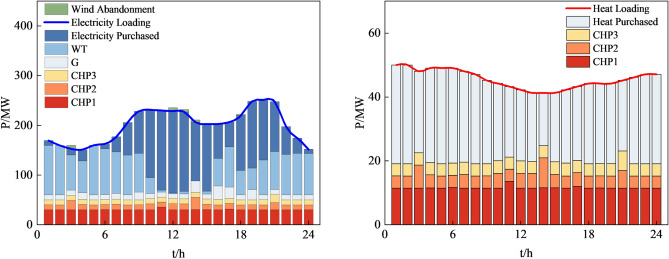
System operation plan obtained in scenarios 1.

**Figure 18:**
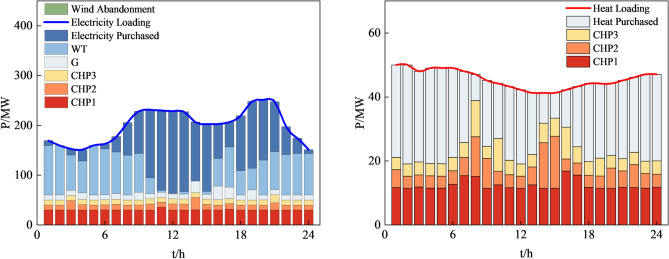
System operation plan obtained in scenarios 2.

**Figure 19:**
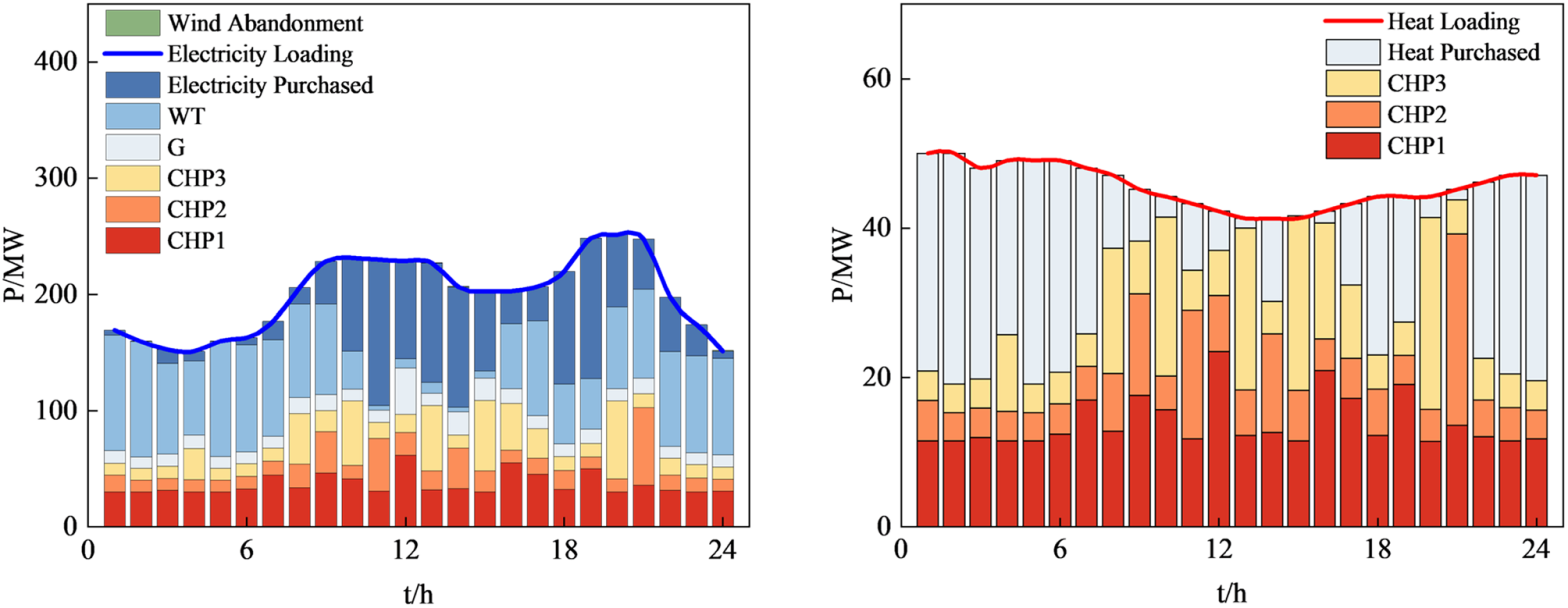
System operation plan obtained in scenarios 3.

Table 10 shows the system operating economic costs and carbon emissions under the three scenarios. Figure 16 displays the Pareto front obtained by MOCAHA cruising and also displays the positions of the each final solutions obtained on the Pareto front. The final system operation plans obtained by each scenario are shown in Figs. 17, 18, and 19, respectively.

**Table 10:** Economic costs and carbon emissions under different scenarios.

Scenarios	Cost/$	Carbon emissions (t)
1	115,376.56	17,783.19
2	120,688.98	16,901.68
3	125,780.81	16,466.11

Combining Table 10, Figs. 16, 17, 18, and 19, the following analysis can be made: compared to Scenario 1, Scenario 2 reduced the subjective weights of the economic cost objective and increased the subjective weights of carbon emissions, resulting in a 4.4% increase in economic cost and a 5.2% reduction in carbon emissions in Scenario 2. Similarly, compared to Scenario 1, Scenario 3 purchased more energy from energy suppliers, leading to an 8.2% increase in economic cost and an 8.0% reduction in carbon emissions. Moreover, all three schemes absorbed wind power effectively, enhancing the utilization rate of renewable energy.

Therefore, TOPSIS-SOCW can integrate the experience of dispatch personnel with the information in the solution set, weighing and trading off between the two optimization objectives of economic cost and carbon emissions. This approach not only considers the relative importance of multiple optimization objectives but also incorporates the expertise of professionals, offering effective support for solving the intricate RIES scheduling problems. Furthermore, this method can also meet the diverse operational requirements of RIES, assisting decision-makers in finding an appropriate compromise between multiple optimization objectives, ultimately realizing low-carbon economic operation of the system.

In conclusion, our simulation analyses validate that the proposed method capitalizes on data comprehensively, diminishes the influence of subjective biases, and addresses the diverse operational needs of the system judiciously. Moreover, compared to traditional approaches that transform multi-objective challenges into single-objective ones via weighting methods, our multi-objective optimization algorithms exhibit superior efficacy. This not only achieves better optimization outcomes but also bolsters both the economic and environmental facets of the regional integrated energy system.

## Conclusions

In this paper, we introduced an optimization method for the low-carbon economic operation of regional integrated energy systems, leveraging the multi-objective chaotic artificial hummingbird algorithm. Through simulation research, our key findings include.

1. A day-ahead scheduling model for the regional integrated energy system was developed, accounting for both economic and environmental aspects. This model is adept at synchronizing and fine-tuning the system's total operational costs and carbon emissions, fulfilling the need for multi-objective coordinated optimization during system operation.
2. The multi-objective chaotic artificial hummingbird algorithm excels in addressing the RIES low-carbon economic coordination optimization model. Notably, it has significant advantages in reducing both economic costs and carbon emissions, with average improvements of 26.60% and 11.13% respectively. The algorithm's iterative convergence results in rapid generation of diverse and spread Pareto front solutions uniformly.
3. Upon establishing the Pareto frontier, our proposed TOPSIS-SOCW effectively harnesses the objective characteristics of the solution set. This integration, coupled with the experiential judgment of dispatchers, facilitates the identification of an optimal compromise solution. This approach ensures that the ultimate dispatch plan not only aligns with the diverse operational requirements of the system but also maintains a high level of objectivity.

The simulation results underscore our method's superior performance in optimization results, convergence efficiency, and solution diversity. Notably, there's an 8.8% reduction in system operational economic costs and a 14.2% drop in carbon emissions.

In our future research endeavors, our primary focus will be on seamlessly integrating diverse energy storage systems. We aim to enhance the system's capacity to incorporate renewable sources and transition towards ultra-reliable clean energy alternatives, such as geothermal and nuclear. Moreover, we plan to explore advanced strategies, including multi-agent technology and clustering techniques, to optimize the scheduling of RIES. This initiative is intended to further improve the overall effectiveness of system optimization. Our overarching ambition is to shape a regional integrated energy system that is both economically viable and environmentally friendly, while also bolstering security measures.

## Data Availability

The datasets generated during and/or analysed during the current study are available from the corresponding author on reasonable request.
